# Sensomics-assisted identification of key aroma components contributing to the characteristic scent of vacuum-extracted *Jin Guanyin* tea hydrolat and its aroma enhancement potential

**DOI:** 10.1016/j.fochx.2025.103106

**Published:** 2025-10-01

**Authors:** Bang-Ming Tang, Jie-Qiong Wang, Xin Meng, Fang Wang, Jian-Xin Chen, Jun-Feng Yin, Yong-Quan Xu

**Affiliations:** a Tea Research Institute Chinese Academy of Agricultural Sciences, State Key Laboratory for Tea Plant Germplasm Innovation and Resource Utilization, 9 South Meiling Road, Hangzhou 310008, China; b College of Horticulture, Fujian Agriculture and Forestry University, Fuzhou 350002, China

**Keywords:** Sensomics, Aroma components, Vacuum extraction, *Jin Guanyin* tea hydrolat, Aroma enhancement potential

## Abstract

Tea hydrolat is an innovative value-added tea product with natural tea aroma. Nevertheless, the dearth of research has hindered its industrial development and commercialization. Therefore, this study is the first to investigate the *Jin Guanyin* Oolong tea hydrolat under vacuum extraction. Sensory evaluation and volatile analysis revealed that low-temperature, short-duration extraction (optimally 50 °C for 40 min) better preserved aroma quality. The 40 aroma components were detected by gas chromatography-olfactometry, and subsequently jasmine lactone, benzeneacetaldehyde, methyl salicylate, hexanal, benzaldehyde, indole, jasmone, methyl heptenone, *β*-ionone, (*Z*)-3-hexen-1-ol, and 1-penten-3-one were identified as the key aroma components of the tea hydrolat based on the aroma recombination and omission tests. Hydrolat scenting demonstrated excellent aroma-enhancing efficacy in tea, achieving up to a 20.69 % increase in key aroma components. These findings indicated the substantial potential for tea hydrolat application in increasing the added value of tea aroma in industry.

## Highlights

• Optimal JGY hydrolat extraction was at low temperature-short duration (50 °C, 40 min).
• Extraction kinetic models for key volatiles were successfully established.
• Key fresh floral components in JGY hydrolat were identified (e.g., jasmine lactone).
• JGY hydrolat could enhance fresh floral, fruity, and sweet aromas of tea leaves.

## Graphical abstract

**Figure:**
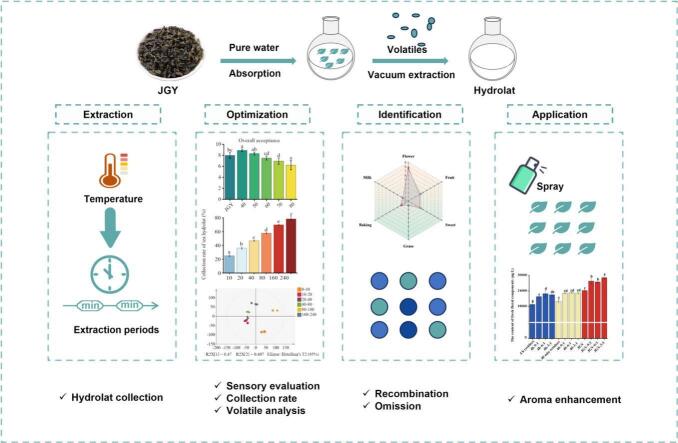

## 1 Introduction

Tea, one of the most important beverages in the world, is renowned not only for its pleasant flavor but also for its multifaceted health benefits, such as lowering blood lipids, blood sugar, anti-inflammatory and antioxidant effects (Wang, Fu, et al., 2022; Zhang et al., 2023). Tea aroma is an important factor affecting the quality of tea (Yang et al., 2013). The aroma composition of tea serves as a critical determinant of quality*,* directly influencing market valuation (Zeng et al., 2020), and acting as an important indicator for distinguishing the flavor characteristics and grades of oolong tea (Zeng et al., 2023).

As new tea gardens continue to appear and tea production increase continuously, thousands of tons of the tea leaves are discarded (Liang et al., 2024). According to the China Tea Production and Marketing Situation Report, China's domestic tea production in 2023 exceeded its annual output by 568,000 tons, representing a 17.01 % increase from the previous year's yield. This substantial growth, amounting to 39.11 % more than the 408,300-ton surplus recorded in 2022, indicates a notable expansion in China's tea production capacity. Comprehensive tea processing is essential for enhancing tea resource utilization efficiency and scaling the tea industry (Cheng, 2018; Liu & H., 2019). Consequently, developing value-added tea products is essential. Up to now, functional components (e.g., tea polyphenols and theanine) have been extensively extracted and utilized in developing high-value tea products, such as tea beverages, functional tea foods, and tea-based health products (Liu & H., 2019). However, natural tea aroma products remain scarce in the commercial market.

Tea hydrolat, an aromatic water co-produced during steam distillation (Meng, Wang, Fu, et al., 2024; Li et al., 2020), preserves the authentic aromatic profile of tea (Xu, Li, et al., 2024) and offers advantages over plant essential oils, such as higher yield, lower cost and greater safety (D'Amato et al., 2018; Long et al., 2023). It has potential applications in food flavoring, beverage formulation, antimicrobial daily chemicals, and clinical therapeutics (Cao, Fu, et al., 2021; Long et al., 2023). Recent studies have begun profiling volatiles in hydrolats from various teas, such as Wuyi rock tea (Fan et al., 2015), cinnamon tea (Xu, Li, et al., 2024), Pu-erh tea (Shi et al., 2023), and jasmine tea (Liu et al., n.d.). However, these earlier studies have not systematically investigated extraction processes, accurately quantified absolute volatile concentrations, or elucidated the release dynamics of key aroma-active compounds.

Oolong tea, especially popular for its strong floral and fruity notes (Guo, 2022; Wang et al., 2025), includes *Jin Guanyin* (JGY)—a variety known for sweet floral aroma from Fujian Province, China (Zheng et al., 2019; Zheng et al., 2022). Sensomics provides a methodological framework for analyzing the sensory quality differences, and is widely applied to characterize tea flavor profile (Cao, Fu, et al., 2021; Wang et al., 2024). The integration of sensomics with SPME-GC/MS provides more comprehensive insights into tea flavor chemistry (Cao, Zhang, et al., 2021). Therefore, this study aimed to characterize the sensory evaluation and aroma quality for JGY hydrolat, optimize the vacuum extraction parameters, identify key aroma-active compounds through conducting gas chromatography-olfactometry (GC-O), aroma recombination and omission experiments, and further verify its aroma enhancement potential. This study opens up new avenues for the development of innovative value-added tea products for the food and flavor industry.

## 2 Materials and methods

### 2.1 Samples and chemicals

JGY was purchased from Anxi Liu Jinlong Tea Co in October 2024. RE5298A rotary evaporator, Shanghai Yarong Biochemical Company; ACS-6 analytical balance, Water Company, USA; HW-CL-1 collector-type constant-temperature magnetic stirring bath, Zhengzhou Changcheng Science, Industry and Trade Co. Aroma standards were presented in Table S1.

**Pre-treatment**: JGY tea (30 g) were placed a spinning flask, and 90 mL of purified water were added according to the ratio of 1:3 (*m/v*), samples were mixed well and placed at room temperature for 60 min, during which the samples were shaken every 20 min to make the tea fully and evenly absorb water.

**Extraction at different temperatures**: The conditions of rotary evaporation were set as follows: a rotation speed of 45 rpm, a vacuum degree 30 mbar, a gradient temperature of 40, 50, 60, 70, 80 °C, and a processing time of 1 h.

**Extraction at different time periods:** Conditions of spinning and evaporation were set as follows: a rotation speed of 45 rpm, a vacuum degree of 30 mbar, a temperature of 50 °C, and a gradient time of 10, 20, 40, 80, 160, 240 min. Under the above set-up conditions, the condensed tea hydrolat in the extraction time intervals of 0–10, 10–20, 20–40, 40–80, 80–160, 160–240 min were collected using collector bottles respectively and stored at 4 °C until use.

### 2.2 Sensory evaluation of JGY hydrolat

The sensory evaluation was done by eight assessors, including four males and four females, from the Tea Research Institute, Chinese Academy of Agricultural Sciences (Hangzhou, China). Before the sensory evaluation, all assessors provided written informed consent form. The study protocol was approved by institution. A 25 mL of the sample was taken in a 50 mL glass vial and gently shaken before sniffing to diffuse the aroma. Sensory qualities were scored in six aroma properties: floral, fruity, sweet, roasted, grassy, and overall acceptability. Aroma intensity was assessed using a 10-point scale: 0–2 for “very weak”, 2–4 for weak, 4–6 for “neutral”, 6–8 for “strong”, 8–10 for “very strong” (Wang et al., 2024), the overall acceptability was scored using a similar process.

### 2.3 Calculation of the hydrolat collection rate and total volatile components

The collection rate was calculated with reference to *Osmanthus* hydrolat (Meng, Wang, Fu, et al., 2024) using the following formula:

$$C_{1}=\frac{\mathrm{\Delta V}\times 100\%}{V}$$

where C_1_ represents the Collection rate (%); ΔV represents the volume (mL) of collected tea hydrolat; V represents the volume (mL) of water added.

The formula for calculating the total volatile components was as follows:

$$\mathrm{Mv}=\mathrm{\Delta V}*C_{2}/1000$$

where M_v_ represents the mass (μg) of volatile components; ΔV represents the mass (mL) of tea hydrolat collected; C_2_ represents the volatile component content (μg/L).

### 2.4 Analysis of volatile components

#### 2.4.1 Aroma volatiles extraction by SPME

**JGY tea:** A 0.5 g of JGY tea was accurately transferred into a 20 mL headspace vial, 5 mL boiling water and 10 μL of ethyl decanoate (internal standard, 10 μg/mL) was added, the headspace vial was rapidly capped. Equilibrium was maintained at 60 °C for 5 min. The head-space volatiles were then absorbed for 55 min using a divinylbenzene/carboxen/polydimethylsiloxane [50/30 μm, stable flex (2 cm)] coating fiber (Supelco, Inc., Bellefonte, PA, USA). Subsequently, the volatiles were desorbed at 250 °C for 5 min in the GC–MS injector. Then, desorbed the fiber 5 min at 250 °C and analyzed by gas chromatography-olfactometry/ mass spectrometry (GC-O/MS).

**JGY hydrolat:** A 5.0 mL of tea hydrolat was accurately transferred into a 20 mL headspace vial, and NaCl was added at a mass content of 0.35 g/mL, 10 μL of ethyl decanoate (internal standard, 10 μg/mL) was added, the headspace vial was rapidly capped. The other treatments were same as those for JGY tea.

#### 2.4.2 GC–MS analysis component

An Agilent 6890 GC interfaced with an Agilent HP 5975 MSD ion trap MS (Wilmington, DE) fitted with a DB-5MS capillary column (30 m × 250 μm × 0.25 μm) was used for volatile component analysis. The GC conditions were as follows: Inlet temperature, 250 °C; carrier gas, high purity helium (99.999 %); flow rate, 1.0 mL/min. The temperature program was as follows: 40 °C for 2 min, increased to 96 °C at 2.5 °C/min, held for 2 min, then increased to 126 °C at 3.5 °C/min, held for 2 min, and increased to 230 °C at 6 °C/min, held for 5 min. The MS analysis was performed at 70 eV in EI mode over a mass range of 40–400 *m/z* using an ion source temperature of 230 °C.

The volatile components were identified using the National Institute of Standards and Technology (NIST) library 98 L mass spectral search program and an internally created GC–MS data analysis program (Wang, Zeng, et al., 2022). The components were identified using the MS library and retention index. In this study, an internal standard method was used to calculate the relative content of the aroma components with reference to the internal standard content and peak area.

### 2.5 Kinetic analysis of aroma components

The first-order kinetic model was fitted using the BoxLucas1 model for the mass of the mass of total aroma components at different extraction times.

$$Q_{t}=Q_{e}\left(1-\exp\left(-\mathrm{kt}\right)\right)$$

Q_t_ represents the mass of the tea hydrolat aroma components at time t, μg; t represents the extraction time, min; k represents the reaction rate constant; Q_e_ represents the final mass of the tea hydrolat aroma components, μg.

Secondary kinetic modeling equation was as follows:

Qt=Qt^2kt/1+Qekt

Q_t_ represents the mass of the tea hydrolat aroma components at time t, μg; t represents the extraction time, min; k represents the quasi-secondary rate constant; Q_e_ represents the final mass of the tea hydrolat aroma components, μg.

### 2.6 GC-O analysis, aroma recombination and omission experiments

#### 2.6.1 GC-O analysis

The samples were divided into two groups (stuffy and fresh floral) according to the sensory evaluation results. The stuffy group included 60, 70, and 80 °C for 1 h and 50 °C for 40–240 min tea hydrolat samples; fresh floral group included 40 and 50 °C for 1 h and 50 °C for 0–40 min samples. The GC-O analysis of aroma components of JGY tea hydrolat was performed by five trained assessors (two males and three females), and each sample was tested twice by each assessor. The tea hydrolat assessors are tea experts who have obtained senior-level professional qualifications in tea sensory evaluation. Before the formal experiment, the assessors group needed to undergo 60 h of training (2 h per day). The training content included triangle testing to correctly identify different aroma types and intensities (Zhu et al., 2024). Firstly, each assessor was well-trained for>60 h during three months in order to get familiar with the different odor descriptions and odor strengths by using a series of standard solutions with different concentrations (Zhu et al., 2018). Subsequently, the assessors were familiarized with the GC-O technique and conducted the GC-O analysis of diverse types of tea samples for 30 h during the final month prior to the actual GC-O analysis. The direct intensity analysis was adapted to analyze the aroma characteristics and odor intensities of aroma components. The aroma intensity (AI) of odor active components was rated on a scale ranging from 0 to 4, as outlined in the study by Yao et al. (2025), with slight modifications. The scale was categorized as follows: 0 was “not sensed,” 1 was “faintly sensed,” 2 was “sensed but faintly recognized,” 3 was “clearly sensed and recognized,” and 4 was “strongly sensed and recognized. The final result was determined by at least two panelist detecting similar detecting similar sensory descriptions of odors and consistent intensity levels during the same time period (Huang et al., 2024). The column (HP-INNOWAX column (60 m × 250 μm × 0.25 μm)) temperature program for GC-O analysis was consistent with that of GC–MS analysis, and the aroma extract was split between the Olfactory Detection Port (ODP3, Gerstel, Germany) and MS with 1:1 proportion. The temperature of the sniffing port was 180 °C and the temperature of the transfer line was maintained 250 °C. The carrier gas of GC-O analysis was high purity nitrogen (99.99 %).

#### 2.6.2 Aroma recombination

All the aroma components with AI >3 identified by GC-O were accurately added to pure water according to the content calculated by external standard method (combined with internal standard method calibration) to obtain the aroma recombination model. The pH of the water was approximately neutral (6.5–7.0) and was not artificially adjusted, nor was the ionic strength modified, to best mimic the simplified matrix of a tea infusion and avoid unintended sensory interactions. The samples extracted at 50 °C for 40 min were used as the control group, and the aroma of the recombination group was scored in 6 attributes: floral, fruity, sweet, grassy, roasted and milk-like. The review was conducted by 20 professionally trained evaluators. Similarity was calculated using cosine similarity (Du et al., 2025), Eq:

$$\text{Similarity}=\frac{\sum_{i=1}^{6}A_{i}\cdot B_{i}}{\sqrt{\sum_{i=1}^{6}A_{i}^{2}}\cdot \sqrt{\sum_{i=1}^{6}B_{i}^{2.}}}$$

A_i_ and B_i_ are the aroma intensity values of the tea hydrolat and the recombination solution, respectively.

#### 2.6.3 Omission test

The key contributing components were determined by systematically omitting certain key aroma substances from the recombination model of tea hydrolat according to the method of Wang et al. (2023) and Liu et al. (2023) According to ISO 4120: 2004 (GB/T 12311–2012) standard, the contribution of each component was evaluated by conducting triangle test. This test aimed to identify which specific aroma components were essential for creating the characteristic aroma profile of the sample.

### 2.7 Application of JGY tea hydrolat for tea scenting

**Preparation of tea hydrolat:** The hydrolat was collected at 50 °C for 40 min, and the other conditions were the same as those described in Section 2.2.

**Scented tea:** The hydrolat was sprayed on tea samples (JGY, 40 min residues and 4 h residues, simulating high, medium, and low aroma intensities, respectively) in the ratio of 1:3, 1:6, 1:9 (hydrolat: tea, *m/m*) ratio and then baked till the moisture content was lower than 5 %.

### 2.8 Statistical analysis

IBM SPSS Statistics (version 23.0, SPSS Inc., Chicago, IL) was performed for analysis of variance (*P* < 0.05), and Origin (version 2024, Origin Lab Corp., Northampton, MA, USA) was used for plotting. SIMCA 14.1 (Umetrics AB, Umea, Sweden) was utilized for chemometrics analysis. All experiments were conducted in triplicate.

## 3 Results and analysis

### 3.1 Aroma quality of JGY tea

**Fig. 1:**
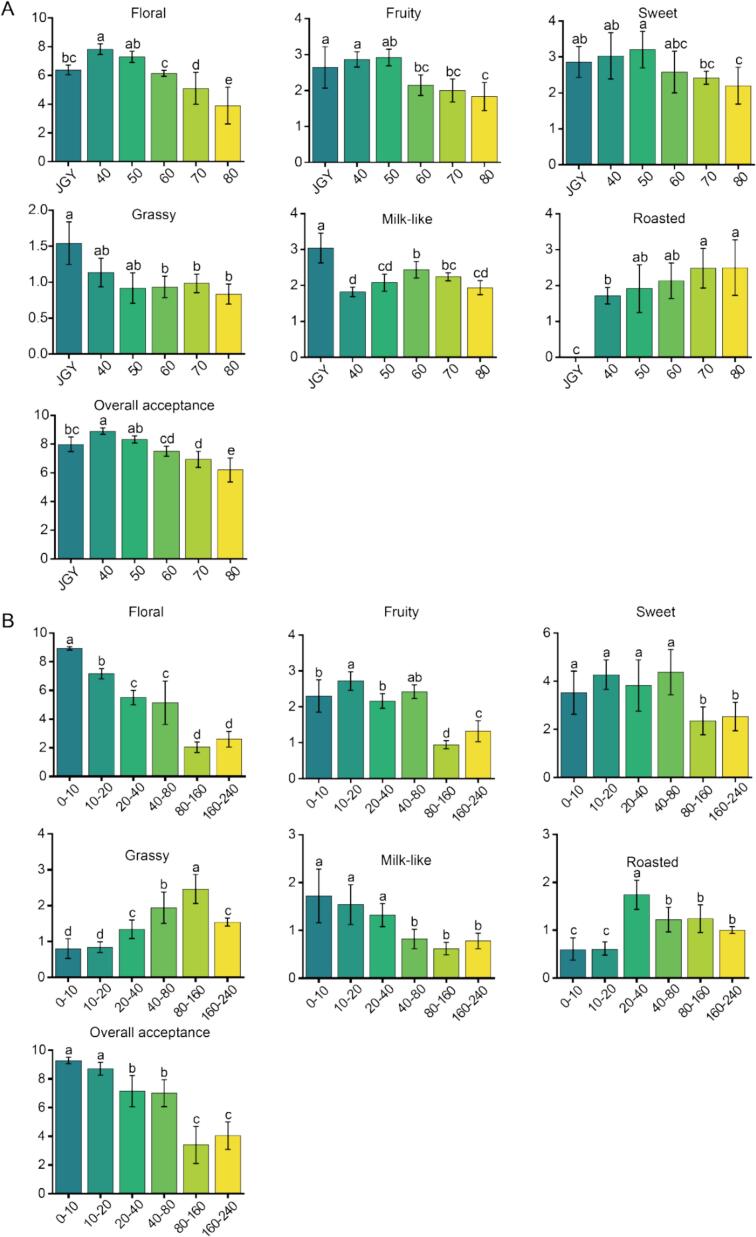
Sensory quality of characteristic aroma of tea hydrolat. (different lowercase letters indicate significant differences between mean values the same column (*p* < 0.05). X-axis: samples; X-axis: scores of sensory evaluations. A: The sensory evaluation results of tea hydrolat at different extraction temperature. B: The sensory evaluation results of tea hydrolat at different extraction time.

Floral, fruity and sweet were recognized as the aroma characteristics of JGY oolong tea (Huang et al., 2024; Zheng et al., 2022). In this research, the aroma profile of JGY tea was characterized by pronounced floral note (Table S2), complemented by secondary fruity, sweet, milk-like, and grassy notes (Fig. 1A).

**Fig. 2:**
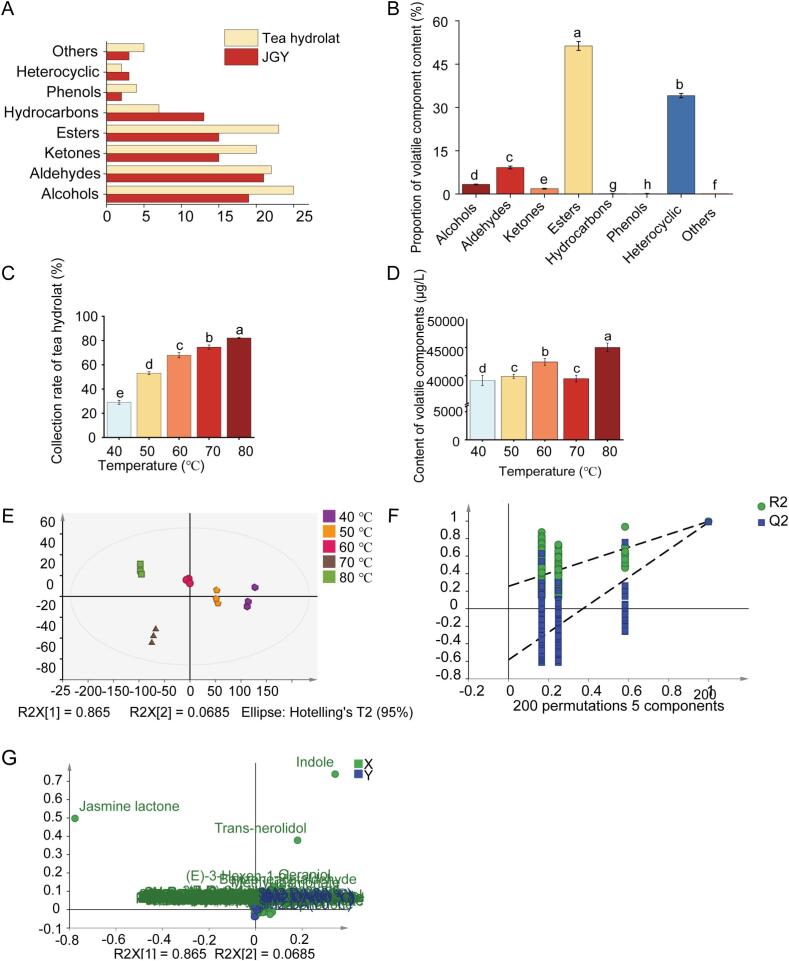
Effect of different extraction temperature on the quality of tea hydrolat. (different lowercase letters indicate significant differences between mean values the same column (*p* < 0.05). A: Types of volatile components in JGY and tea hydrolat; B: Proportion of volatile components content in tea. C: Tea hydrolat collection rate; D: Volatile component content of tea hydrolats; E: PLS-DA score scatter plot; F: Validation of PLS-DA model; G: Load scatter plot.

According to GC–MS analysis, 91 volatile components (Table S3) were detected in JGY tea, including 21 aldehydes, 19 alcohols, 15 ketones, 15 esters, 13 hydrocarbons, 2 phenols, 3 heterocyclic components and 3 others (Fig. 2A).

Esters were the main aroma components of JGY oolong tea, consistent with the research (Huang et al., 2024), constituting 51.29 % of total content (Fig. 2B). Key esters included jasmine lactone, methyl jasmonate, and methyl salicylate, with jasmine lactone (sweet, floral, fruity notes) exhibiting the highest content (18,644.50 μg/L). Heterocyclic components include indole, 2-pentylfuran and 2-ethylfuran, constituting 34.08 % of total content, in which indole (floral) was the most abundant (12,617.13 μg/L). Hydrocarbons and phenols were minor components, accounting for 0.13 % and 0.02 %, respectively (Fig. 2B).

### 3.2 Optimization of tea hydrolat under different extraction temperatures

Plant hydrolat retains the characteristic aroma profiles of plants (Long et al., 2023), however, extraction efficiency and aroma quality are critically influenced by temperature during vacuum distillation (Meng, Wang, Fu, et al., 2024). To optimize thermal parameters for tea hydrolat production, the extraction of samples at different temperature gradients (40–80 °C) was systematically evaluated.

#### 3.2.1 Sensory evaluation

The quality of the tea hydrolat obtained from the extraction was dominated by floral aroma, and relative to the tea raw material, it had a certain roasted aroma. With the increase of temperature, the floral, fruity and sweet aroma were weakened, and the roasted aroma increased (Fig. 1A). The tea hydrolat exhibited dominant floral notes at lower temperatures (40–50 °C), with optimal sensory harmony (> 8.0) and a distinct fresh floral profile. Elevated temperatures (≥ 60 °C) obviously attenuated floral, fruity, and sweet attributes while enhanced roasted notes, ultimately resulting in a decline in fresh floral aroma and an increase of stuffy aromas (Table S2). It was hypothesized that lower temperatures (40 and 50 °C) could minimize structural degradation of thermolabile floral components, therefore preserving the characteristic fresh floral aroma. While elevated temperatures (> 60 °C) created a process similar to roasting, which accelerated moisture evaporation, leading to a decrease in floral characteristics and an increase in roasted odor.

#### 3.2.2 Analysis of the extraction rate of tea hydrolat

The collection rate of tea hydrolat under different temperature conditions was presented in Fig. 2C. The collection rate of tea hydrolat was the lowest at 40 °C, which was only 29.03 %, and the collection rate gradually increased with the increase of temperature, surpassing 50 % within the range of 50–80 °C and peaking at 82.19 % under 80 °C. These findings confirmed temperature as a critical operational parameter governing tea hydrolat extraction efficiency.

#### 3.2.3 Aroma quality analysis

Tea hydrolat effectively preserved most of the volatile profile of JGY while containing more abundant in alcohols, esters, ketones and aldehydes. A total of 108 volatile components were detected in the five tea hydrolat samples of JGY (Table S3), including 25 alcohols, 22 aldehydes, 23 esters, 20 ketones, 7 hydrocarbons, 4 phenols, 2 heterocyclic components, and 5 others. Compared to JGY tea (Fig. 2A), the tea hydrolat was richer in alcohols, esters, ketones and aldehydes, and had relatively fewer hydrocarbons. Water-soluble components are the main composition of hydrolat (Jakubczyk, 2021). Alcohols, esters, and simple aldehydes possessed higher water solubility and were more easily dissolved in water and distilled from tea. Thermal extraction dynamics significantly influenced the content of aroma components. The lowest content of aroma components, 39160.44 μg/L, was found in the tea hydrolat extraction at 40 °C. Subsequently, the total content of aroma components exhibited an increasing trend with the increase of temperature, and the highest content was extracted at 90 °C which was 45,030.64 μg/L (Fig. 2D). Heterocyclic components were the most abundant aroma components in tea hydrolat (Table S3), with indole exhibiting the highest abundance (21,655.94 μg/L at 40 °C). The content of indole decreased significantly with increasing temperature. The esters were the second abundant volatile components, mainly include jasmine lactone, methyl jasmonate and methyl salicylate. The proportion of ester components gradually increased from 15.54 % to 41.70 % with the increase of extraction temperature. Among the esters, jasmine lactone with sweet, floral and fruity characteristics accounted for the highest amount, with a maximum content of 17,980.80 μg/L at 80 °C.

To explore the differences in aroma composition of tea hydrolat obtained at different temperatures of collection, cluster analysis of 108 aroma components was conducted (Fig. S1A). According to the clustering results, the aroma components can be divided into two major categories (Fig. S1A), the first category contained 27 components, mainly include jasmine lactone, methyl jasmonate, phenylethyl alcohol and others, exhibiting an increasing trend of the content with extraction temperature, these components are more easily leached at high extraction temperature. Another category contained 81 components, mainly include indole, jasmone, benzeneacetaldehyde and others, the content declined at elevated extraction temperature, the reduction of these components was responsible for the weakening of tea hydrolat aroma. These components failed to effectively condense in the extract due to their greater sensitivity to temperature during high-temperature extraction. High temperature decreased the abundance of floral and grassy aroma components in tea (Gao et al., 2022). Elevated temperature of the extraction lead to degradation and generation of other components (Cao, Zhang, et al., 2021), such as the contents of *β*-ionone, *α*-farnesene, *trans*-nerolidol, indole and benzeneacetaldehyde and hexanal decreased significantly at high temperatures.

To further explore the differences in aroma components of JGY tea hydrolat at different temperatures of collection, the characteristic differential aroma components of the five groups of samples were further analyzed using the partial least squares discriminant analysis (PLS-DA) model. The model was equipped with a fitting parameter of R^2^X = 0.998, R^2^Y = 0.994, and Q^2^ = 0.985 (Fig. 2E), which verified its good robustness. In addition, the parameters of the valid model (R^2^ = 0.244, Q^2^ = −0.576) indicated its absence of overfitting (Fig. 2F). And the loading diagram was presented in Fig. 2G. The metabolites located on the perimeter of the plot could be identified as important differential components for distinguishing differences between treatment groups (Wang et al., 2024). A total of 11 key differential substances with VIP > 1 was screened, including jasmine lactone, methyl jasmonate, indole, *trans*-nerolidol, benzeneacetaldehyde, jasmone, (*E*,*E*)-2,4-heptadienal, (*Z*)-3-hexen-1-ol, 3,5-octadien-2-one, hexanal and geraniol, which were the key differential components under different temperature conditions (Table S3). Among the 11 components, the contents of jasmine lactone and methyl jasmonate, which exhibited floral and sweet aromas (Ho et al., 2015), increased with elevated temperature. And the content of other 9 components decreased with elevated temperature, including 3 grassy aroma components and 6 floral, fruity and sweet aroma components. Jasmine lactone and methyl jasmonate have floral and sweet aroma (Ho et al., 2015), the content of these two components increased with elevated temperature. Notably, the content of jasmine lactone, reached the peak at 80 °C with a content of 17,980.80 μg/L, while methyl jasmonate had the highest content of 737.50 μg/L at 60 °C. The extraction of volatile components in vacuum distillation was closely related to the boiling point (Chalier et al., 2024), volatile substances having higher boiling points need more energy for vaporization (Zheljazkov et al., 2014). Although the extraction was carried out under reduced pressure, which could lower the boiling points of the components (Chalier et al., 2024), the energy need for the effective leaching of jasmine lactone may still not be reached at low temperatures. This may explain the higher content of jasmine lactone in JGY tea hydrolat extracted at high temperature.

Overall, extraction temperature exerted a significant impact on the quality and functional properties of JGY tea hydrolat. Sensory evaluation revealed an inverse correlation between temperature and aroma quality. The tea hydrolat obtained from 40 °C extraction had the best aroma quality, but the collection rate was lower. The tea hydrolat obtained from 60, 70 and 80 °C extraction exhibited significant weakness in aroma and appeared a stuffy flavor. In contrast, tea hydrolat obtained from 50 °C extraction maintained good sensory quality, and reached an extraction rate of 53.09 %. Therefore, considering the quality of tea hydrolat and the collection rate, 50 °C was considered the optimum parameter for tea hydrolat extraction.

### 3.3 Optimization of tea hydrolat under different extraction time periods

The quality of hydrolat transformed significantly at different extraction periods (He et al., 2020). In order to clarify the effect of extraction time periods on the quality of tea hydrolat, based on the results of tea hydrolat extraction at different temperatures, and synthesizing the collection rate and the sensory quality of tea hydrolat, 50 °C was chosen as the subsequent extraction temperature. Subsequently, extraction experiments at different extraction time periods were conducted at 50 °C.

#### 3.3.1 Sensory evaluation

The sensory evaluation of tea hydrolat was exhibited at Fig. 1B. With the extension of extraction time, the overall acceptance of the tea hydrolat of JGY exhibited a decreasing trend, and there was a slightly increase in 160–240 min. 0–10 min and 10–20 min tea hydrolat exhibited obvious fresh floral aroma, and after 40 min, the fresh floral aroma of the tea hydrolat was weakened, while the roasted aroma raised, with the appearance of stuffy odor (Table S2). After 80 min, the floral, sweet, fruity and overall acceptability decreased significantly, which led to a decrease in the aroma quality of the tea hydrolat.

#### 3.3.2 Analysis of the extraction rate of tea hydrolat

The collection rate of tea hydrolat exhibited a time-dependent extraction profile characterized by an initial rapid accumulation phase followed by progressive saturation. As illustrated in Fig. S2, the increase in extraction rate gradually slowed down over time. The collection rate of tea hydrolat increased rapidly in the first 40 min, reaching a collection of 46.81 %. after 40 min, the collection rate increased slowly, and the increase of the collection rate from 40 to 240 min (31.49 %) was lower than that from 0 to 20 min (36.17 %), which indicated that the extraction efficiency of tea hydrolat was lower after 40 min.

#### 3.3.3 Aroma quality analysis

A total of 102 volatile components were identified during the six extraction time periods, including 23 alcohols, 23 aldehydes, 19 ketones, 21 esters, 3 phenols, 6 hydrocarbons, 1 heterocyclic component, and 6 others (Table. S3). From the perspective of the total amount of volatile components, the highest was observed in 0–10 min (up to 39,092.57 μg/L), followed by 80–160 min (up to 37,621.46 μg/L), while other samples exhibited no significant differences (Fig. S2D). Through cluster analysis, they could be classified into 2 major classes according to the content dynamics (Fig. S1B). 52 components represented by geraniol, *β*-ionone, and jasmone, etc., exhibiting an increasing trend in content with the extraction. 50 components represented by 1-penten-3-one, benzaldehyde, 3,5-octadien-2-one, etc., exhibiting a decreasing trend in content as the extraction time increased.

To explore the differences in the aroma components of the tea hydrolat at different extraction periods, the characteristic differential aroma components of the samples at six different time periods were further analyzed by conducting the PLS-DA model, with the fitting indices of R^2^X = 0.991, R^2^Y = 0.922, and Q^2^ = 0.691 (Fig. S2E), and the loading diagram was shown in Fig. S2F. Parameters of valid model (R^2^ = 0.361, Q^2^ = −0.763) indicated its absences with of overfitting (Fig. S2G), the intersection of Q^2^ regression line with the vertical axis was <0, which confirmed the reliability of the applied PLS-DA model. 15 key aroma components with VIP > 1 were screened by PLS-DA analysis, these components were the key substances affecting the aroma quality of tea hydrolat at different time periods. Among them, the content of 9 components, including benzeneacetaldehyde, jasmine lactone, (*Z*)-3-hexen-1-ol, (*E*,*E*)-2,4-heptadienal, 3,5-octadien-2-one, methyl heptenone, benzaldehyde, hexanal, and linalool, exhibited a decreasing trend as the extraction proceeded, with linalool and methyl heptenone undetected, after 160 min. Synergistic interactions, including masking effects (benzaldehyde and methyl salicylate) and cumulative amplification (hexanal and benzeneacetaldehyde, linalool and hexanal/benzaldehyde), were observed (Jin et al., 2024), and the depletion of these components were likely to lead to a decrease in the floral, fruity, and sweet aromas of the tea hydrolat.

**Fig. 3:**
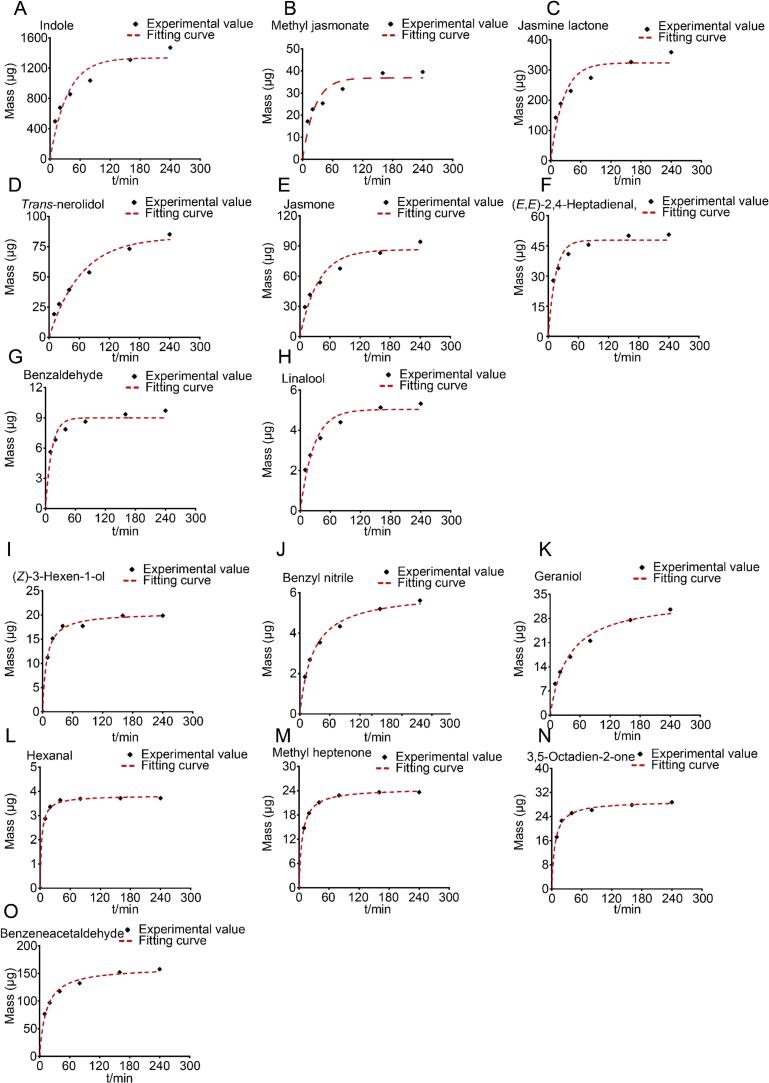
Kinetic fitting of key volatile components. A: Indole; B: Methyl jasmonate; C: Jasmine lactone; D: *Trans*-nerolidol; E: Jasmone; F: (*E*,*E*)-2,4-Heptadienal; G: Benzaldehyde; H: Linalool; I: (*Z*)-3-Hexen-1-ol; J: Benzyl nitrile; K: Geraniol; L: Hexanal; M: Methyl heptenone; N: 3,5-Octadien-2-one; O: Benzeneacetaldehyde. Experimental value: the mass of volatiles in experiments. Fitting curve: the mass of volatiles predicted.

The volatilization kinetics of the volatile components under vacuum extraction conditions were observed (Dong et al., 2023; Pang et al., 2023). Pseudo-first-order and pseudo-second-order kinetic model were widely used in describing solid-liquid extraction processes, especially in tea brewing and volatile compound release (Pang et al., 2023; Xu et al., 2018). To further investigate the leaching pattern of volatile components, the volatilization kinetics of key aroma components during vacuum extraction were systematically analyzed through pseudo-first-order and pseudo-second-order kinetic modeling, with time as the independent variable and total mass as the dependent variable. Among the 15 components, 8 adhered to pseudo-first-order kinetics (R^2^ > 0.90), including indole, methyl jasmonate, jasmine lactone, *trans*-nerolidol, jasmone, (*E,E*)-2,4-heptadienal, benzaldehyde, and linalool, which exhibited near-perfect fits (R^2^ > 0.95). Solvent concentration critically influenced tea extract leaching kinetics. Extraction efficiency peaked at low solvent concentrations but declined as saturation approached (Xu et al., 2018). The leaching rates of 8 target components correlated strongly with the tea matrix-solvent concentration gradient. Initially, elevated solute levels in the tea matrix drove rapid leaching. As extraction progressed, solvent depletion through volatilization concurrent with diminishing tea solute content reduced the concentration gradient, thereby decreasing leaching rates. It was observed from the fitted curves that the mass increase of these 8 components was low after 40 min, and the mass basically reached equilibrium at 160 min (Fig. 3A-H). Conversely, seven components, including (*Z*)-3-hexen-1-ol, benzyl nitrile, geraniol, hexanal, methyl heptenone, 3,5-octadien-2-one and benzeneacetaldehyde, followed pseudo-second-order kinetics (R^2^ > 0.95). At the initial stage of extraction stage, the leaching of these components was faster, probably due to the fact that the components in the outer layer of the raw material were less bound to the raw material and could be easily extracted (Xi et al., 2014), and as the extraction proceeded, the leaching of these components was limited by solubility or adsorption of the tea, and the rate of leaching decreased rapidly. Relative to the 8 volatile components that fit the first-order kinetic model, the mass increase of these seven components decreased more rapidly and took less time to reach equilibrium, with the mass essentially reaching equilibrium at 80 min (Fig. 3I-O). Increasing the pressure can improve the extraction rate of the components (Liu et al., 2024; Xi et al., 2014; Xi et al., 2015). In future experiments, reducing the vacuum and increasing the pressure could be applied to improve the quality of tea hydrolat aroma. Overall, all 15 aroma components exhibited high correlation coefficients (R^2^ > 0.90), indicating a well-fitted model that reliably simulates their release kinetics during extraction.

In conclusion, the extraction dynamics of JGY tea hydrolat were governed by two critical time thresholds: 40 min and 80 min. After 40 min, the yield increase rate declined significantly, accompanied by sensory degradation marked by rising roasted and grassy notes alongside diminished floral intensity and overall acceptability; Beyond 80 min, the system approached extraction equilibrium, with further deterioration in quality-stale odors intensified, while floral, fruity, and sweet attributes decreased. These results demonstrated the superiority of shorter extraction periods (0–40 min), which preserved the character of fresh floral profile while avoiding roasted and coarse odors. Optimized protocols should prioritize this initial phase to balance yield efficiency with premium sensory and functional quality.

### 3.4 Identification of key active aroma components in JGY tea hydrolat

Temperature and time extraction parameters critically influenced the volatile character of JGY tea hydrolat. The results of the sensory evaluation revealed the dominance of a fresh floral aroma, which was attenuated under elevated temperatures (> 60 °C) and prolonged extraction times (> 40 min). To figure out the key fresh floral aroma components, the samples were divided into two groups of stuffy and fresh floral, as listed in Section 2.7.1. The active components of JGY tea hydrolat were determined by GC-O/MS. The 50 °C, 40 min sample, characterized by a fresh floral profile, was served as the control for recombination and omission experiments to identify key odorants.

#### 3.4.1 GC-O analysis

A total of 40 components, including 12 alcohols, 9 aldehydes, 7 ketones, 6 esters, 3 phenols, 1 hydrocarbon, 1 heterocyclic component and 1 other, were identified in the two groups of samples (Table 1). Compared with the samples in the stuffy group, 10 components such as pentanol were only smelled in the “fresh floral” group. 27 components were detected in both the two groups, including 1-penten-3-one, indole, jasmine lactone, benzaldehyde, hexanal, and so on. 23 components in the fresh-flower aroma group had an AI of >3, which contributed significantly to the aroma of JGY tea hydrolat. Indole, methyl jasmonate, benzeneacetaldehyde, jasmine lactone, (*Z*)-3-hexen-1-ol, *trans*-nerolidol, jasmone, (*E*,*E*)-2,4-heptadienal, 3,5-octadien-2-one, geraniol and hexanal, were identified as the key components in the experiments at different extraction temperatures and time periods, notably, their AIs were found to be >3 in the GC-O sniffing. Among them, (*E*,*E*)-2,4-heptadienal was found to exhibit grassy aroma (Huang et al., 2024), (*Z*)-3-hexen-1-ol exhibits grassy, fresh and root-like, and hexanal exhibits a grassy, fresh and apple-like aroma (Kun et al., 2025), the three components contributed to the grassy aroma of the hydrolat.

**Table 1:** Aroma active components and aroma intensity of JGY tea hydrolat.

No. a	RI b (DB-5MS)		Compounds	Odor c	AI d		Identification e
	Calculated	Referenced			Stuffy	Fresh floral	
1	661	682	1-penten-3-one	mushroom	3	3	MS, RI, O, Std
2	762	765	1-pentanol	sweet	–	1	MS, RI, O
3	801	801	hexanal	grassy, herbaceous plant	2	3	MS, RI, O, Std
4	848	855	(*Z*)-3-hexen-1-ol	grassy, leaves	3	3	MS, RI, O, Std
5	898	905	*cis*-4-heptenal	grassy	3	3	MS, RI, O, Std
6	957	962	benzaldehyde	bitter almond	4	3	MS, RI, O, Std
7	969	970	heptanol	ethanol	–	2	MS, RI, O
8	980	980	1-octen-3-ol	asparagus	2	–	MS, RI, O
9	984	986	methyl heptenone	fresh and fruity	2	3	MS, RI, O, Std
10	998	1012	(*E*,*E*)-2,4-heptadienal	grassy herbaceous plant	3	4	MS, RI, O, Std
11	1003	1003	octanal	grass, herbaceous plant	–	2	MS, RI, O
12	1042	1045	benzeneacetaldehyde	floral, hyacinth	4	3	MS, RI, O, Std
13	1056	1060	(*E*)-2-octenal	grassy	3	3	MS, RI, O, Std
14	1060	1066	acetophenone	floral	4	3	MS, RI, O, Std
15	1072	1070	octanol	fruity and fresh	–	2	MS, RI, O
16	1084	1086	linalool oxide II (*trans*-furan)	floral	–	2	MS, RI, O
17	1092	1091	3,5-octadien-2-one	sweet, milk-like	3	4	MS, RI, O, Std
18	1103	1099	linalool	floral, sweet	4	4	MS, RI, O, Std
19	1107	1106	dihydrolinalool	floral, sweet	2	3	MS, RI, O
20	1112	1116	phenylethyl alcohol	floral	2	2	MS, RI, O
21	1137	1144	benzyl nitrile	plastics	–	2	MS, RI, O
22	1139	1142	3-nonen-2-one	fruity	2	3	MS, RI, O, Std
23	1187	1192	methyl salicylate	minty	2	3	MS, RI, O, Std
24	1192	1189	*α*-terpineol	camphor	–	2	MS, RI, O
25	1201	1224	2-propylphenol	fumigate	2	3	MS, RI, O, Std
26	1212	1222	2-amino-benzaldehyde	fruity	2	–	MS, RI, O
27	1254	1255	geraniol	sweet, rose scent	3	3	MS, RI, O, Std
28	1283	1261	*γ*-octanolactone	creamy, coconut	3	2	MS, RI, O
29	1302	1295	indole	floral	4	3	MS, RI, O, Std
30	1305	1317	*trans*-2,4-decadienal	grassy herbaceous	3	1	MS, RI, O.
31	1394	1395	jasmone	jasmine, sweet, fruity	3	3	MS, RI, O, Std
32	1448	1453	*trans*-isoeugenol	floral, carnation	–	2	MS, RI, O
33	1456	1457	*α*-farnesene	woody	2	–	MS, RI, O
34	1479	1486	*β*-ionone	floral, sweet	3	3	MS, RI, O, Std
35	1485	1488	2-phenylethyl 2-ethylbutyrate	sweet, floral	3	1	MS, RI, O
36	1488	1518	jasmine lactone	creamy, floral	2	3	MS, RI, O, Std
37	1491	1497	5-decanolide	creamy, coconut	–	2	MS, RI, O
38	1509	1514	2,4-ditert-butylphenol	fruity	–	2	MS, RI, O
39	1576	1564	*trans*-nerolidol	fruity, grapefruit	3	3	MS, RI, O, Std
40	1640	1638	methyl jasmonate	floral, fruity	2	3	MS, RI, O, Std

a Compounds were ordered with their elution order in DB-5MS column.

b Retention indices were determined using a homologous series of *n*-alkanes (C_7_-C_40_).

c Odor quality was detected at a sniffing port, comparing with aroma attributes recorded in papers.

d AI values indicated mean aroma intensity values of aromatic substances were detected by sniffing, the final result was determined by at least two panelist detecting similar detecting similar sensory descriptions of odors and consistent intensity levels during the same time period (Huang et al., 2024).

e Odorants were identified by comparison of their odor quality, retention indices (RIs) on DB-5MS capillary columns as well as mass spectrometry (MS) data with the data of authentic standard compounds (Std).

Indole, which exhibited floral attributes, is a characteristic aroma component of oolong tea (Guo, 2022; Zeng et al., 2023). Jasmone, geraniol, jasmone lactone, methyl jasmonate, *trans*-nerolidol, benzeneacetaldehyde, 3,5-octadien-2-one exhibited floral, fruity, and sweet aromas, were the significant flavor components of oolong tea (Ho et al., 2015; Huang et al., 2024; Wang et al., 2023; Zheng et al., 2022). Moreover, the five components, benzeneacetaldehyde, geraniol, (*E*,*E*)-2,4-heptadienal, 3,5-octadien-2-one, and hexanal, decreased significantly under elevated temperatures or prolonged extraction times, indicating that these components were the important components responsible for a decrease in the fresh floral aroma of the tea hydrolat.

#### 3.4.2 Aroma recombination and omission tests

Some components lack reliable odor threshold data or may exhibit significant variability due to individual or cultural differences (Kun et al., 2025). In addition to components with OAV > 1, components with OAV < 1 may also interact with other components to affect the overall aroma (Tao et al., 2025), GC-O is effective in identifying aroma-active components in a complex system (Lu et al., 2023), therefore, identification of aroma recombination components was primarily based on GC-O results. The tea hydrolat samples at 50 °C for 40 min were selected as the control group, excluding dihydrolinalool due to commercial unavailability. The aroma components with AI >3 in the fresh floral group were dissolved in water based on the precise quantification results of the external standard method (combined with internal standard method calibration) (Table S4).

**Fig. 4:**
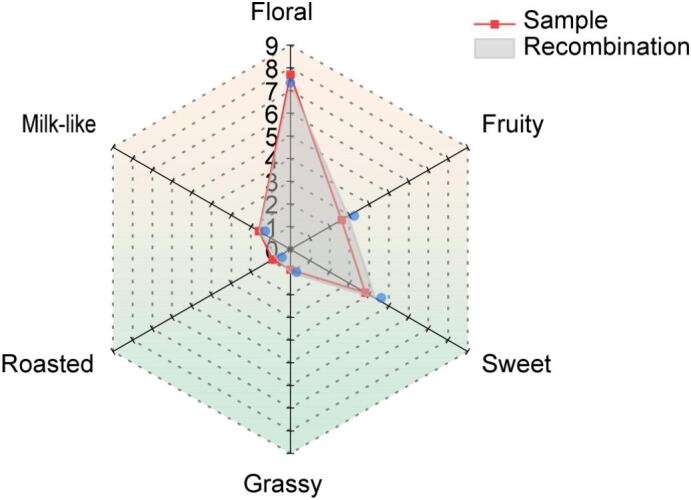
Comparison of aroma quality between recombinant model and tea hydrolat. Sample: tea hydrolat sample. Recombination: the recombination model.

The results of the sensory evaluation, which included floral, fruity, sweet, roasted, grassy, and milk-like aromas, revealed a high degree of similarity (cosine similarity = 0.98) between the recombinant and natural hydrolats (Fig. 4). However, subtle differences emerged: the recombinant model exhibited slightly weaker floral and roasted aromas than the tea hydrolat, and slightly stronger fruity and sweet aromas. The statistical analysis confirmed the absence of significant differences, thereby validating the efficient characterization of the predominant aromas in JGY tea hydrolat.

A triangle sensory test was conducted to determine the impact of specific aroma component on the aroma of tea hydrolat by systematically omitting specific component from the recombined model. The results, as shown in Table 2, revealed that when jasmine lactone, benzeneacetaldehyde, methyl salicylate, hexanal, benzaldehyde, indole, jasmone, methyl heptenone, *β*-ionone, (*Z*)-3-hexen-1-ol, and 1-penten-3-one were missing from the tea hydrolat, significant differences were observed (*P* < 0.05). However, no significant differences were observed when *trans*-nerolidol, methyl jasmonate, geraniol, linalool, (*E*,*E*)-2,4-heptadienal, *trans*-2-octenal, *cis*-4-heptenal, 2-n-propylphenol, acetophenone, 3-nonen-2-one, and 3,5-octadien-2-one were omitted in the model solution. Consequently, jasmine lactone, benzeneacetaldehyde, methyl salicylate, hexanal, benzaldehyde, indole, jasmone, methyl heptenone, *β*-ionone, (*Z*)-3-hexen-1-ol, and 1-penten-3-one were regarded as key aroma components of the fresh floral aroma of JGY tea hydrolat.

**Table 2:** Omission test of tea hydrolat model.

No.	Omitted compounds	Odor	Correct answers/panelists	Significant level
1	jasmine lactone	sweet, floral, fruity	15/20	***
2	benzeneacetaldehyde	floral, hyacinth	15/20	***
3	methyl salicylate	cool, minty	13/20	**
4	hexanal	grassy herbaceous plant	12/20	*
5	benzaldehyde	bitter almond	12/20	*
6	indole	floral	11/20	*
7	jasmone	jasmine, sweet, fruity	11/20	*
8	methyl heptenone	fresh, fruity	11/20	*
9	*β*-ionone	floral, sweet scent	11/20	*
10	(*Z*)-3-hexen-1-ol	grassy, leaves	11/20	*
11	1-penten-3-one	mushroom	11/20	*
12	*trans*-nerolidol	fruity, grapefruit peel	5/20	–
13	methyl jasmonate	floral, fruity	5/20	–
14	geraniol	sweet, rose	8/20	–
15	linalool	floral, sweet scent	10/20	–
16	(*E*,*E*)-2,4heptadienal	green, grassy, herbaceous plant	4/20	–
17	*trans*-2-octenal	grassy	5/20	–
18	*cis*-4-heptenal	grassy	7/20	–
19	2-propylphenol	fumigate	9/20	–
20	acetophenone	floral	7/20	–
21	3-nonen-2-one	fruity	10/20	–
22	3,5-octadien-2-one	sweet, milk-like	6/20	–

Significance: ***: (*p* < 0.001); **: 0.001 ≤ *p* ≤ 0.01; *: 0.01 < p ≤ 0.05; −: (*p* > 0.05).

### 3.5 Application of JGY tea hydrolat for tea scenting

Plant hydrolats have demonstrated significant potential as natural flavoring agents in food applications (Long et al., 2023). Tea leaves possess numerous pore structures and high specific surface area (Xu, Li, et al., 2024), were recognized for their superior adsorption capacity (Shindel et al., 2025; Xu, Ma, et al., 2024), have been effectively utilized to absorb aroma components. Based on these findings, this part investigated the aroma enhancement efficacy of tea hydrolat through a scenting process. The experimental design involved uniform application of tea hydrolat onto tea leaves, leveraging their inherent adsorption properties.

#### 3.5.1 Sensory evaluation of scented tea

**Fig. 5:**
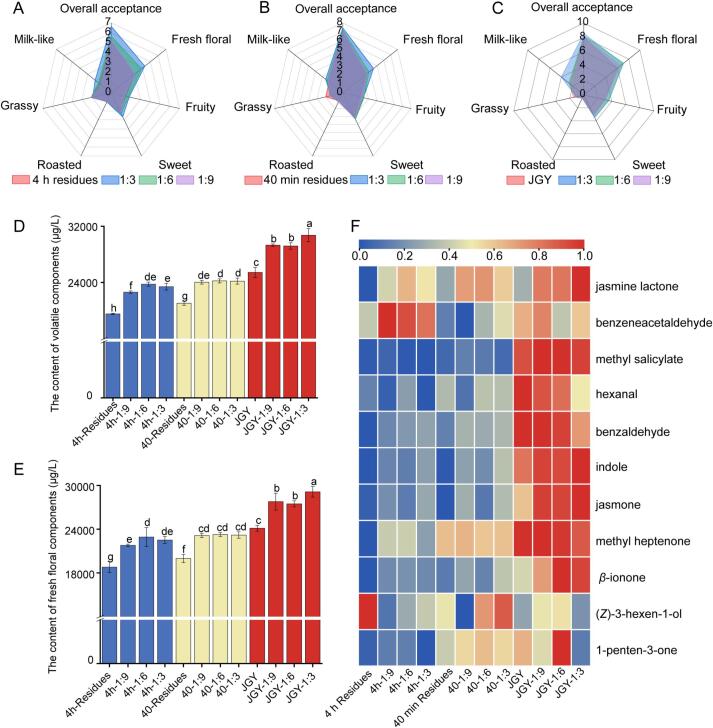
Sensory and volatile analysis of scented tea. (different lowercase letters indicate significant differences between mean values the same column (*p* < 0.05). A: Sensory evaluation of 4 h residues and scented tea; B: Sensory evaluation of 40 min residues and scented tea; C: Sensory evaluation of JGY and scented tea; D: The total content of volatile components; E: The total content of key fresh floral components; F: Heatmap of key fresh floral components.

Sensory evaluation confirmed the aroma-enhancing effect of tea hydrolat (Fig. 5A-C). After the treatment of tea hydrolat, the fresh floral, fruity, sweet and milk-like aromas of the tea increased, while the grassy aroma slightly decreased, roasted aroma remained stable. Compared to the control tea material (ck), the most significant improvements of the scores and improvement proportions of sensory evaluation were highest under optimal conditions: fresh floral aroma peaked in sample 4 h-1:3 (increasing from 3.00 to 4.30, + 43.33 %), fruity aroma in 40–1:6 (2.00 to 2.43, + 23.33 %), sweet aroma in 4 h-1:3 (2.00 to 2.67, + 33.33 %), and milk-like aroma in JGY-1:3 (2.50 to 4.00, + 60 %). Conversely, the largest reduction in grassy aroma occurred in 40–1:3 (decreasing from 2.00 to 1.40, − 30 %). In the meanwhile, with the increase of the spraying ratio of tea hydrolat, the aroma of scented tea was enhanced, and the best fragrance effect were observed under the 1:3 treatment in the three kinds of tea raw materials.

#### 3.5.2 Volatile components of scented tea

There were significant differences in the aroma composition of the scented teas made from different hydrolat-tea ratios and tea raw materials. A total of 81 volatile components were identified in the scented tea (Table S5), including 18 alcohols, 17 aldehydes, 14 ketones, 12 esters, 13 hydrocarbons, 2 phenols, 3 heterocyclic components and 2 others. The total content of volatile components of the three tea raw materials was significantly different (Fig. 5D), with the highest content of 25,427.25 μg/L in JGY tea, and the lowest content of 19,530.94 μg/L in the 4 h tea residues. Compared with the tea raw materials, the volatile component content of the scented tea exhibited an increasing trend, verifying the aroma enhancement effect of tea hydrolat. When treated at a 1:3 ratio, both 40-min tea residues and JGY tea exhibited significantly higher volatile compound content than other ratios, exhibiting increases of 14.90 % and 20.85 % respectively compared to the control (ck). Excessive water would block various pores in the tea (Tang, 2000), which leads to a decrease in aroma absorption. In the 4 h samples, there was no significant difference between the 1:3 and 1:6 ratios of the treated samples, probably because the previous extraction process affected the porosity of the tea and weakened the adsorption properties of the tea.

To explore the scenting effect of tea hydrolat, the content of 11 key fresh floral components identified by recombination and omission tests was analyzed (Fig. 5E), the highest content of the key components was observed in JGY-1:3 sample, and the lowest content was observed in 4 h residues. The total content of fresh floral components increased significantly after scenting, and exhibited an increase trend at higher tea-hydrolat ratio, with highest improvements of 20.69 % in JGY-1:3 (Fig. 5E), implying the aroma enhancement effect of tea hydrolat. Among the 11 key fresh floral components, (*Z*)-3-hexen-1-ol and hexanal contributed to the fresh and grassy odor, and their contents declined after the treatment of tea hydrolat (Fig. 5F). It is a common process for tea to adsorb aroma from high concentration to low concentration (Xu, Li, et al., 2024). The content of (*Z*)-3-hexen-1-ol and hexanal in tea hydrolat was lower than that of tea, which led to a decrease of the content in the tea. Moreover, the decomposition and transformation at high temperature was the reason for the weakening of grassy aroma in scented tea. The content of jasmine lactone (sweet, floral, fruity), benzeneacetaldehyde (sweet, floral), methyl salicylate (minty), benzaldehyde (bitter almond), indole (floral), jasmone (jasmine, fruity), methyl heptenone (fresh, fruity), *β*-ionone (floral, sweet), and 1-penten-3-one (mushroom) increased after the tea hydrolat was scented, which accounted for the increase of the fresh floral, fruity, sweet, and milk-like flavors of scented tea. In addition, correlation analysis (Fig. S3) revealed a positive correlation between key volatile compounds, including jasmine lactone, methyl salicylate, hexanal, benzaldehyde, indole, jasmone, methyl heptenone, *β*-ionone, and 1-penten-3-one, and fresh floral, fruity, sweet, and milk-like aromas. However, these compounds exhibited a negative correlation with roasted and grassy notes. Hexanal, which is typically associated with green olfactory qualities, exhibited a significant negative correlation with the presence of a grassy aroma. This unanticipated finding may be attributed to the complex aromatic context of the tea samples, wherein fresh floral, fruity, and sweet notes predominate the sensory profile. Within this complex matrix, the grassy character of hexanal is likely masked by more potent odorants such as methyl salicylate and other components, illustrating the occurrence of olfactory masking effects. Experimental verification of this conjecture will be carried out in the future.

Therefore, tea hydrolat treatment significantly enhanced aroma quality, with both hydrolat-tea ratio and tea raw materials critically influencing aroma enhancement efficacy. JGY had the best adsorption effect for aroma, and the aroma-enhancement effect of tea hydrolat was optimal when added in the ratio of 1:3.

## 4 Conclusions

This study first systematically investigated the effects of extraction temperature and time on the aroma profile of tea hydrolat under vacuum conditions. Comparative analysis revealed that the content of alcohols, ketones, and aldehydes was higher in tea hydrolat than in tea raw materials. Although the higher extraction temperatures (> 60 °C) improved the yield efficiency, the floral and fruity aroma decreased and undesirable stuffy odor appeared. Subsequently, it was found that 0–40 min was the optimal extraction period, which preserved the fresh floral aroma of tea hydrolat.

Kinetic analysis exhibited that the changes in the 8 key aroma components such as indole, jasmine lactone, jasmone follow a first-order kinetic model (R^2^ > 0.95), while the other 7 aroma components such as geraniol, hexanal, and methyl heptanone follow a second-order kinetic model (R^2^ > 0.99). These models could be applied for the prediction and optimization of tea hydrolat aroma quality. Through recombination and omission experiments, 11 aroma components were confirmed as significant contributors to the fresh floral aroma in tea hydrolat. Finally, by spraying the tea hydrolat onto samples treated with different aroma intensities, it was found that the fresh floral, fruity, and sweet aromas of the tea leaves could be effectively enhanced. These findings provide a mechanistic foundation for developing tea hydrolat products and understanding the volatilization patterns of tea-scented components. However, the extraction efficiency and aroma characteristics of tea hydrolat in this study may still be constrained by raw material characteristics (such as cultivation variety and pore structure) and equipment limitations. Moreover, as a tea hydrolat product, tea hydrolat has lots of value-added potentials such as flavor harmonization of beverages and tea aroma spray formulation. All of these will be taken into consideration in the future.

## CRediT authorship contribution statement

**Bang-Ming Tang:** Writing – original draft, Software, Methodology, Investigation, Formal analysis, Data curation. **Jie-Qiong Wang:** Writing – review & editing. **Xin Meng:** Methodology, Formal analysis, Data curation. **Fang Wang:** Validation. **Jian-Xin Chen:** Validation. **Jun-Feng Yin:** Visualization. **Yong-Quan Xu:** Writing – review & editing, Supervision, Project administration, Funding acquisition.

## Ethics statement

Before conducting the sensory tests described in this study, panel members from the Tea Research Institute of the Chinese Academy of Agricultural Sciences were fully informed about the test procedures, requirements, and potential risks. All participants provided written informed consent prior to their involvement. Additionally, formal approval to carry out the sensory tests was obtained from the institution.

## Declaration of competing interest

The authors declare that they have no known competing financial interests or personal relationships that could have appeared to influence the work reported in this paper.

## Data Availability

Data will be made available on request.

## Appendix B Supplementary data

Supplementary material Image 1

## References

[bb0010] Cao Q.-Q., Fu Y.-Q., Wang J.-Q., Zhang L., Wang F., Yin J.-F., Xu Y.-Q. (2021). Sensory and chemical characteristics of Tieguanyin oolong tea after roasting. Food Chemistry: X.

[bb0015] Cao X., Zhang F., Cai G., Liu J., Hong Z.Z.P., Sun S., X. H (2021). Analysis and reconstitution of characteristic aromas in tie Guanyin tea based on sensory-oriented separation and its application in cigarette flavoring. Acta Tabacaria Sinica.

[bb0020] Chalier P., Martinez-Lopez B., Lacour M.A., Rigou P. (2024). Extraction of turpentine essential oil from pinus pinaster ait: Comparison of yield and composition between conventional- or microwave assisted-hydro-distillation and vacuum distillation. Sustainable Chemistry and Pharmacy.

[bb0025] Cheng Z. (2018). Innovation and development of China tea industry in new era. Journal of Agriculture.

[bb0030] D’Amato S., Serio A., López C.C., Paparella A. (2018). Hydrosols: Biological activity and potential as antimicrobials for food applications. Food Control.

[bb0035] Dong Z., Liu H., Guo X., Xiong H., Yang B., Xu B. (2023). Volatilization kinetics and thermodynamic stability of antimony sulfide under vacuum conditions. Vacuum.

[bb0040] Du Z., Zhou Y., Guo S., Dong Y., Xu Y., Yu X. (2025). Triterpenoid saponins in tea plants: A spatial and metabolic analysis using UPLC-QTOFMS, molecular networking, and DESI-MSI. Food Chemistry.

[bb0045] Fan J., Yang W., Lian Y., Huo X., Cao X., Hu J., Zhang X. (2015). The preparation, analysis and application of flavor extracts from Wuyi rock tea. Flavour Fragrance Cosmetics.

[bb0050] Gao Y., Cao Q.-Q., Chen Y.-H., Granato D., Wang J.-Q., Yin J.-F., Xu Y.-Q. (2022). Effects of the baking process on the chemical composition, sensory quality, and bioactivity of Tieguanyin oolong tea. Frontiers in Nutrition.

[bb0055] Guo X. (2022). Characterization of the aroma profiles of oolong tea made from three tea cultivars by both GC–MS and GC-IMS. Food Chemistry.

[bb0060] He J., Fu M., Zou X., Xiao Y. (2020). Study on volatile components and antioxidant ability of essential oil and hydrosol from rosmarinus officinalis at different distillation periods. China Condiment.

[bb0065] Ho C.-T., Zheng X., Li S. (2015). Tea aroma formation. Food Science and Human Wellness.

[bb0070] Huang H., Zheng Y., Hu C., Wu Q., Yang Y., Ou X., Zhao M., Sun (2024). Analysis of key aroma components of three representative oolong tea varieties by stir bar sorptive extraction combined with gas chromatography-olfactory-mass spectrometry. Food Science.

[bb0075] Jakubczyk K. (2021). Plant hydrolates – Antioxidant properties, chemical composition and potential applications. Biomedicine & Pharmacotherapy.

[bb0080] Jin L., Lian X., Chen L., Lei Y., Li J., Yang Z., Li D. (2024). Characteristic aroma analysis and interaction study of key aroma components of chuanhong congou black tea. European Food Research and Technology.

[bb0085] Kun J. (2025). Characterization of potential aroma components in five aroma types of green tea using the sensomics approach. LWT.

[bb0095] Liang S., Gao Y., Granato D., Ye J., Zhou W., Yin J., Xu Y. (2024). Pruned tea biomass plays a significant role in functional food production: A review on characterization and comprehensive utilization of abandon-plucked fresh tea leaves. Comprehensive Reviews in Food Science and Food Safety.

[bb0100] Liu P.-P., Feng L., Xu Y.-Q., Zheng L., Yin P., Ye F., Gui A.-H., Wang S.-P., Wang X.-P., Teng J., Xue J.-J., Gao S.-W., Zheng P.-C. (2023). Characterization of stale odor in green tea formed during storage: Unraveling improvements arising from reprocessing by baking. LWT.

[bb0105] Liu, X., Wu, H., Liu, X., & Luo, L., Analytical comparison on flavor components of colorless tea extracts prepared from two different methods. *Journal of Food Safety and Quality*, *11*(13), 4371–4378. doi:10.19812/j.cnki.jfsq11-5956/ts.2020.13.043.

[bb0110] Liu Y., Feng X., Gao T., Pan Y., Lv H., Chen M., Shen Y., Zhu W., Yao Y., He L., Gong S., Fan F., Chu Q., Song C., Chen P. (2024). Advancement and challenges in tea brewing: The dynamic principles, influencing factors, innovative processing technologies and pollutants. Trends in Food Science & Technology.

[bb0115] Liu Z., H. (2019). The development process and trend of Chinese tea comprehensive processing industry. Journal of Tea Science.

[bb0120] Long D., Hong P., Li B., Xie G., Cheng X., Li J., Xu Y., Jiang H., Jiang H. (2023). Progress of processing technology and biological activity of tea and plant aromatic water. China Tea Processing.

[bb0125] Lu C., Zhang Y., Zhan P., Wang P., Tian H. (2023). Characterization of the key aroma components in four varieties of pomegranate juice by gas chromatography-mass spectrometry, gas chromatography-olfactometry, odor activity value, aroma recombination, and omission tests. Food Science and Human Wellness.

[bb0135] Meng X., Wang F., Fu C.-H., Zeng L., Chen Z.-H., Du Q., Feng Z.-H., Yin J.-F., Xu Y.-Q. (2024). Effect of osmanthus hydrolat on the aroma quality and volatile components of osmanthus black tea. Food Chemistry: X.

[bb0150] Pang J., Kong L., Yang B., Yang Z., Wu H., Zhao W., Zhang J., Xu J., Xu B. (2023). Kinetics of indium and indium–tin soldering materials in vacuum volatilization. Journal of Materials Research and Technology.

[bb0155] Shi H., Zhang Y., Yang D., Wang C., Zhang J. (2023). Analysis of volatile compounds and optimization of extraction conditions in perfume of Pu-erh tea condensed reflux tea. Journal of Tianjin Agricultural University.

[bb0160] Shindel B., Harms C., Wang S., Dravid V. (2025). Brewing clean water: The metal-remediating benefits of tea preparation. ACS Food Science & Technology.

[bb0165] Tang Y. (2000). Aroma absorbing and keeping mechanism of tea. Journal of tea.

[bb0170] Tao M., Guo W., Liang J., Liu Z. (2025). Unraveling the key cooked off-flavor components in thermally sterilized green tea beverages, and masking effect of tea raw material baking. Food Chemistry.

[bb0175] Wang B.-H., Huang P.-H., Lo C.-Y., Chang W.-C. (2025). Metabolomic analysis elucidates the dynamic changes in aroma components and the milk aroma mechanism across various portions of tea leaves during different stages of oolong tea processing. Food Research International.

[bb0180] Wang J.-Q., Fu Y.-Q., Chen J.-X., Wang F., Feng Z.-H., Yin J.-F., Zeng L., Xu Y.-Q. (2022). Effects of baking treatment on the sensory quality and physicochemical properties of green tea with different processing methods. Food Chemistry.

[bb0185] Wang J.-Q., Tang B.-M., Gao Y., Chen J.-X., Wang F., Yin J.-F., Xu Y.-Q. (2024). Impact of heat treatment on the flavor stability of Longjing green tea beverages: Metabolomic insights and sensory correlations. Food Research International.

[bb0190] Wang L., Wu L., Xiang D., Huang H., Han Y., Zhen P., Shi B., Chen S., Xu Y. (2023). Characterization of key aroma components in aged qingxiangxing baijiu by comparative aroma extract dilution analysis, quantitative measurements, aroma recombination, and omission studies. Food Chemistry.

[bb0195] Wang S., Zeng T., Zhao S., Zhu Y., Feng C., Zhan J., Li S., Ho C.-T., Gosslau A. (2022). Multifunctional health-promoting effects of oolong tea and its products. Food Science and Human Wellness.

[bb0200] Xi J., He L., Yan L. (2015). Kinetic modeling of pressure-assisted solvent extraction of polyphenols from green tea in comparison with the conventional extraction. Food Chemistry.

[bb0205] Xi J., Yan L., He L. (2014). Pressure-dependent kinetic modeling of solid–liquid extraction of the major green tea constituents. Separation and Purification Technology.

[bb0210] Xu L., Ma C., Chen X., Du Q., Song C., Li X., Xu Y.-Q. (2024). Theories and applications of tea residue adsorbing aroma components: A review. Beverage Plant Research.

[bb0215] Xu X., Li C., Li A., Zheng K., Pan J., Hou M. (2024). Analysis of the volatile components in the pure dew of cinnamon tea. Fujian Agricultural Science and Technology.

[bb0220] Xu Y.-Q., Ji W.-B., Yu P., Chen J.-X., Wang F., Yin J.-F. (2018). Effect of extraction methods on the chemical components and taste quality of green tea extract. Food Chemistry.

[bb0230] Yang Z., Baldermann S., Watanabe N. (2013). Recent studies of the volatile components in tea. Food Research International.

[bb0240] Yao X., Li Y., Tang J., Yu J., Zhang Y., Wan X., Zhai X. (2025). Characterization of cooked off-flavor volatile sulfur-containing compounds in green tea and their thermal inhibition via (−)-epigallocatechin gallate. Food Chemistry.

[bb0245] Zeng L., Fu Y.-Q., Liu Y.-Y., Huang J.-S., Chen J.-X., Yin J.-F., Jin S., Sun W.-J., Xu Y.-Q. (2023). Comparative analysis of different grades of tieguanyin oolong tea based on metabolomics and sensory evaluation. LWT.

[bb0250] Zeng L., Zhou X., Su X., Yang Z. (2020). Chinese oolong tea: An aromatic beverage produced under multiple stresses. Trends in Food Science & Technology.

[bb0255] Zhang L., Xu Y., Liu Z. (2023). Tea: From historical documents to modern technology. Molecules.

[bb0260] Zheljazkov V.D., Astatkie T., Schlegel V. (2014). Hydrodistillation extraction time effect on essential oil yield, composition, and bioactivity of coriander oil. Journal of Oleo Science.

[bb0265] Zheng Y., Hu Q., Wu Z., Bi W., Chen B., Hao Z., Wu L., Ye N., Sun Y. (2022). Volatile metabolomics and coexpression network analyses provide insight into the formation of the characteristic cultivar aroma of oolong tea (camellia sinensis). LWT.

[bb0270] Zheng Y., Wang P., Chen X., Sun Y., Yue C., Ye N. (2019). Transcriptome and metabolite profiling reveal novel insights into volatile heterosis in the tea plant (camellia sinensis). Molecules.

[bb0280] Zhu J., Liu X., Lin Y., Ke Q., Niu Y., Zhang J., Yang E., Shen T., Sun Z., Xiao Z. (2024). Unraveling the characteristic chestnut aroma compounds in MeiTanCuiYa green tea and their interaction mechanisms with broad-spectrum olfactory receptors using molecular docking. LWT.

[bb0285] Zhu Y., Lv H.-P., Shao C.-Y., Kang S., Zhang Y., Guo L., Dai W.-D., Tan J.-F., Peng Q.-H., Lin Z. (2018). Identification of key odorants responsible for chestnut-like aroma quality of green teas. Food Research International.

