# Supplementary material for: Sensomics-assisted identification of key aroma components contributing to the characteristic scent of vacuum-extracted Jin Guanyin tea hydrolat and its aroma enhancement potential
Source: Food Chem X. 2025 Oct 1;31:103106. doi: 10.1016/j.fochx.2025.103106 (PMC12537572; doi:10.1016/j.fochx.2025.103106)
Supplement: Supplementary file 1 — Supplementary material [file mmc1.docx]

**Figure captions**

**Fig. S1.** Cluster analysis of volatile components. A: Volatiles of tea hydrolat at different extraction temperature; B: Volatiles of tea hydrolat at different extraction time. 1 indicates the category of volatiles that demonstrate an increase in trend with increasing extraction temperature or time. 2 indicates the category of volatiles that demonstrate a decrease in trend with increasing extraction temperature or time.

**Fig. S2.** Effect of different extraction time on the quality of tea hydrolat. (different lowercase letters indicate significant differences between mean values the same column (*p* < 0.05). A: Extraction rate of tea hydrolat; B: The content of volatile components; C: Scatter plot of PLS-DA score; D: Scatter plot of loading; E: Validation of PLS-DA model.

**Fig. S3.** Heatmap of the correlation between key fresh floral components and sensory attributes.

**Table captions**

**Table S1.** Relevant standard information of volatile compounds.

**Table S2.** Description of JGY and tea hydrolat aroma quality.

**Table S3.** Volatiles content (μg/L) of JGY and tea hydrolat under different extraction conditions.

**Table S4.** The quantitative results of key active components in 50 ℃, 40 min tea hydrolat sample.

**Table S5.** Changes in the content of volatiles under different scenting treatments.


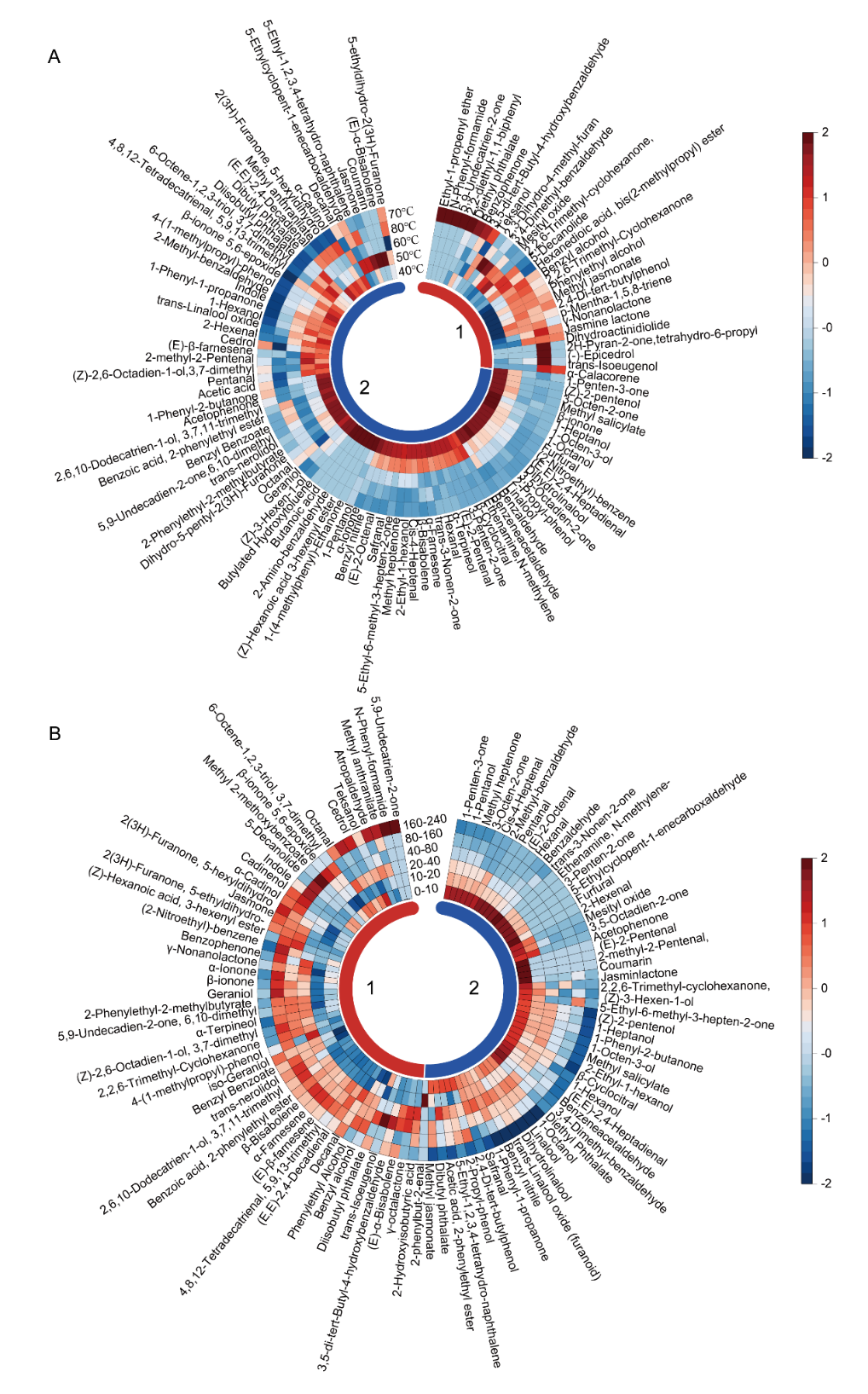


**Fig. S1** Cluster analysis of volatile components. A: Volatiles of tea hydrolat at different extraction temperature; B: Volatiles of tea hydrolat at different extraction time. 1 indicates the category of volatiles that demonstrate an increase in trend with increasing extraction temperature or time. 2 indicates the category of volatiles that demonstrate a decrease in trend with increasing extraction temperature or time.


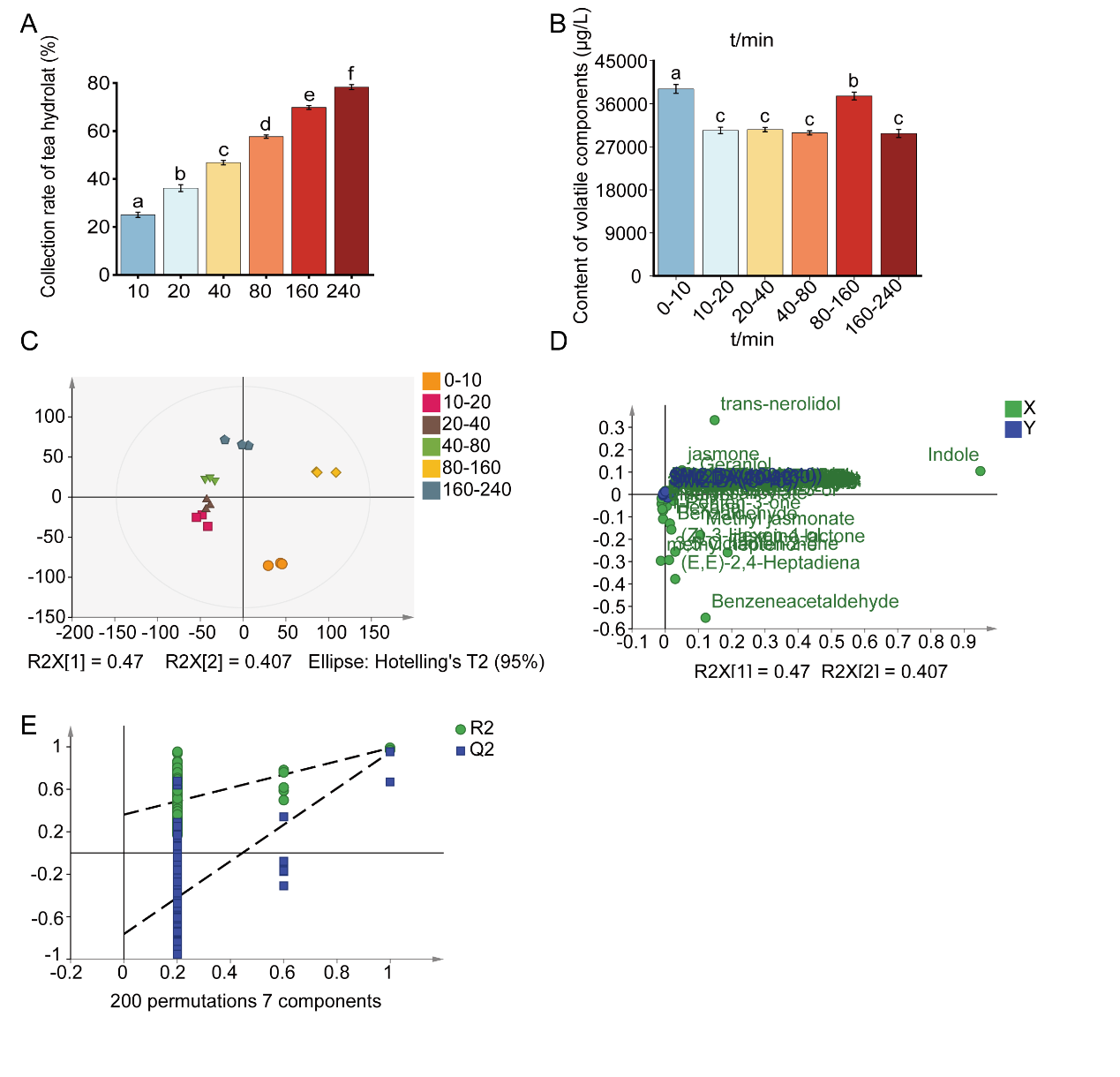


**Fig. S2** Effect of different extraction time on the quality of tea hydrolat. (different lowercase letters indicate significant differences between mean values the same column (*p* < 0.05). A: Extraction rate of tea hydrolat; B: The content of volatile components; C: Scatter plot of PLS-DA score; D: Scatter plot of loading; E: Validation of PLS-DA model.

**
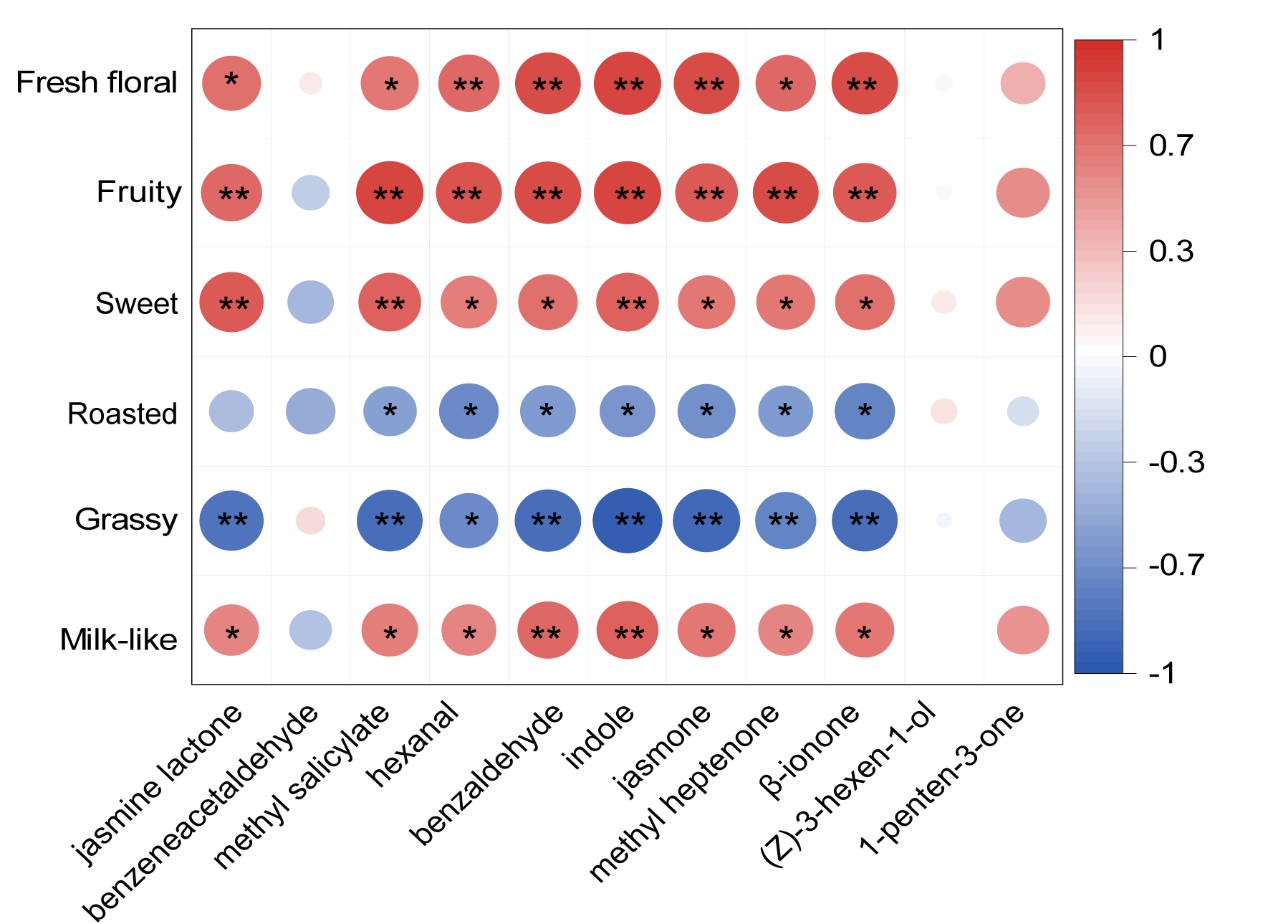
**

**Fig. S3.** Heat map of the correlation between key fresh floral components and sensory attributes.

**Table S1** Relevant standard information of volatile compounds.

| Chemicals | Related information | | | |
| --- | --- | --- | --- | --- |
|  | Purity | Brand | City | Country |
| Benzaldehyde | ≥ 99% | Macklin | Shanghai | China |
| (*Z*)-3-Hexen-1-ol | 98% | Macklin | Shanghai | China |
| *Trans*-3-Nonen-2-one | > 95% | Macklin | Shanghai | China |
| 2-Propylphenol | ≥ 98% | Macklin | Shanghai | China |
| Jasmine lactone | 97% | Macklin | Shanghai | China |
| Methyl jasmonate | 98% | Macklin | Shanghai | China |
| Jasmone | 98% | Macklin | Shanghai | China |
| *Trans*-2-octenal | > 95% | Macklin | Shanghai | China |
| *Cis*-4-heptenal | ≥ 95% | Aladdin | Shanghai | China |
| Hexanal | 97% | Aladdin | Shanghai | China |
| Methyl heptenone | 98% | Aladdin | Shanghai | China |
| Acetophenone | > 99.5% | Aladdin | Shanghai | China |
| Indole | 99% | Aladdin | Shanghai | China |
| 1-Penten-3-one | 97% | Aladdin | Shanghai | China |
| Linalool | > 96% | TCI | Shanghai | China |
| Ethyl decanoate | > 99% | TCI | Shanghai | China |
| *β*-Ionone | 97% | Yuanye | Shanghai | China |
| Trans-nerolidol | ≥ 95% | Yuanye | Shanghai | China |
| Benzeneacetaldehyde | 95% | Yuanye | Shanghai | China |
| Methyl salicylate | ≥ 98% | Yuanye | Shanghai | China |
| γ-Octanolactone | > 98% | Yuanye | Shanghai | China |
| (*E*, *E*)-2,4-Heptadienal | > 90% | Bidepharm | Shanghai | China |
| Geraniol | > 98% | Bidepharm | Shanghai | China |
| 3,5-Octadien-2-one | ≥ 95% | Anpel | Shanghai | China |

Other reagents: NaCl (analytical purity, purity ≥ 99%) was purchased from Sinopharm Chemical Reagent Co., Ltd; ethanol (chromatographic grade, purity ≥ 99%) was purchased from Shanghai Huishi Co. (Shanghai, China). Purified water was purchased from Hangzhou Wahaha Group Co (Hangzhou, China).

**Table S2** Description of JGY and tea hydrolat aroma quality.

| Sample | Description |
| --- | --- |
| JGY | Floral, pure |
| 40 ℃, 1 h | Fresh floral, pure |
| 50 ℃, 1 h | Fresh floral |
| 60 ℃, 1 h | With fresh floral, slightly stuffy |
| 70 ℃, 1 h | Light fresh floral, slightly stuffy |
| 80 ℃, 1 h | Light fresh floral, stuffy |
| 50 ℃, 0-10 min | Heavy fresh floral |
| 50 ℃, 10-20 min | Fresh floral |
| 50 ℃, 20-40 min | With fresh floral |
| 50 ℃, 40-80 min | Slightly fresh floral, slightly stuffy |
| 50 ℃, 80-160 min | Coarse, stuffy |
| 50 ℃, 160-240 min | With coarse, stuffy |

**Table S3.** Volatiles content (μg/L) of JGY and tea hydrolat under different extraction conditions.

| RT | Compounds | Category | Content (μg/L) | | | | | | | | | | | |
| --- | --- | --- | --- | --- | --- | --- | --- | --- | --- | --- | --- | --- | --- | --- |
|  |  |  | JGY | 40 ℃ | 50 ℃ | 60 ℃ | 70 ℃ | 80 ℃ | 0-10 min | 10-20 min | 20-40 min | 40-80 min | 80-160 min | 160-240 min |
| 2.67 | Ethyl-1-propenyl ether | other | - | - | - | - | 0.05 | - | - | - | - | - | - | - |
| 2.83 | Acetic acid | other | 0.10 | - | - | - | - | - | - | - | - | - | - | - |
| 2.93 | 1-Penten-3-one | ketone | 11.92 | 21.29 | 12.73 | 7.43 | 7.58 | 5.00 | 93.17 | 58.99 | 51.99 | 31.07 | 21.53 | 5.64 |
| 3.14 | Pentanal | aldehyde | 0.75 | 0.30 | 0.32 | 0.12 | 0.18 | - | 0.66 | 0.26 | 0.14 | - | - | - |
| 3.18 | 2-Ethylfuran | heterocyclic compound | 0.18 | - | - | - | - | - | - | - | - | - | - | - |
| 3.58 | Ethenamine, N-methylene | other | - | 0.07 | 0.07 | - | - | - | 0.18 | 0.07 | - | - | - | - |
| 3.89 | 3-Penten-2-one | ketone | - | 0.40 | 0.28 | - | - | - | 0.34 | 0.14 | - | - | - | - |
| 4.01 | Tiglic aldehyde | aldehyde | 0.10 | - | - | - | - | - | - | - | - | - | - | - |
| 4.27 | (*E*)-2-Pentenal | aldehyde | 0.44 | 1.02 | 0.73 | - | - | - | 0.70 | - | - | - | - | - |
| 4.60 | 1-Pentanol | alcohol | 0.49 | 2.56 | 1.40 | 1.14 | 1.28 | 1.29 | 3.24 | 1.86 | 1.49 | 0.89 | 0.55 | 0.17 |
| 4.66 | (*Z*)-2-pentenol | alcohol | - | 1.56 | 0.69 | - | - | - | 1.33 | 1.05 | 1.05 | 0.76 | 0.59 | - |
| 5.03 | 2,3-dihydro-4-methyl-furan | heterocyclic compound | - | - | - | 0.36 | - | 0.11 | - | - | - | - | - | - |
| 5.39 | Mesityl oxide | ketone | - | 1.17 | 1.57 | 1.50 | 1.24 | 1.39 | 1.92 | 1.36 | - | - | - | - |
| 5.51 | Hexanal | aldehyde | 244.03 | 108.44 | 79.39 | 20.79 | 30.82 | 28.21 | 127.12 | 49.21 | 29.04 | 5.45 | 2.04 | 0.32 |
| 6.50 | 2-Methyl-2-pentenal | aldehyde | 0.64 | 0.49 | 0.59 | 0.36 | 0.42 | 0.32 | 0.87 | - | - | - | - | - |
| 6.56 | Furfural | aldehyde | - | 0.43 | 0.29 | 0.27 | 0.20 | 0.19 | 0.32 | 0.14 | - | - | - | - |
| 7.39 | 2-Hexenal | aldehyde | 0.83 | 0.95 | 0.88 | 0.59 | - | - | 1.10 | 0.29 | - | - | - | - |
| 7.48 | (*Z*)-3-Hexen-1-ol | alcohol | 403.82 | 524.39 | 309.86 | 295.58 | 183.43 | 253.53 | 497.38 | 391.29 | 269.74 | 0.00 | 195.53 | 0.00 |
| 7.64 | Ethylbenzene | hydrocarbon | 0.09 | - | - | - | - | - | - | - | - | - | - | - |
| 8.03 | m-Xylene | hydrocarbon | 0.16 | - | - | - | - | - | - | - | - | - | - | - |
| 8.10 | 1-Hexanol | alcohol | - | 0.93 | 1.05 | 0.71 | - | 0.45 | 0.73 | 0.50 | 0.44 | 0.39 | - | - |
| 8.16 | 2-Hydroxyisobutyric acid | other | - | - | - | - | - | - | - | - | 0.38 | 0.28 | 0.23 | - |
| 8.94 | Styrene | hydrocarbon | 2.34 | - | - | - | - | - | - | - | - | - | - | - |
| 8.95 | 2-Heptanone | ketone | 0.38 | - | - | - | - | - | - | - | - | - | - | - |
| 9.29 | Butyl acrylate | ester | - | - | - | - | - | - | - | - | - | - | - | - |
| 9.39 | *Cis*-4-Heptenal | aldehyde | 2.53 | 3.27 | 2.10 | 0.95 | 0.00 | 0.00 | 1.87 | 0.92 | 0.68 | 0.00 | 0.00 | 0.00 |
| 9.52 | Heptaldehyde | aldehyde | 0.92 | - | - | - | - | - | - | - | - | - | - | - |
| 10.63 | Methyl hexanoate | ester | - | - | - | - | - | - | - | - | - | - | - | - |
| 12.45 | Benzaldehyde | aldehyde | 113.35 | 219.01 | 170.25 | 142.82 | 127.60 | 118.23 | 248.83 | 120.70 | 107.70 | 78.70 | 65.85 | 48.04 |
| 13.08 | 1-Heptanol | alcohol | 0.47 | 2.39 | 1.26 | 1.05 | 0.97 | 0.95 | 1.64 | 1.35 | 1.35 | 1.06 | 0.79 | 0.28 |
| 13.66 | 1-Octen-3-ol | alcohol | 1.45 | 6.68 | 4.54 | 3.77 | 3.51 | 3.29 | 6.17 | 4.52 | 4.51 | 3.27 | 2.13 | 0.67 |
| 13.87 | Methyl heptenone | ketone | 246.10 | 550.78 | 490.68 | 373.67 | 277.91 | 273.62 | 654.54 | 364.71 | 279.62 | 176.87 | 73.59 | - |
| 14.05 | 2-Pentylfuran | heterocyclic compound | 0.79 | - | - | - | - | - | - | - | - | - | - | - |
| 14.46 | Butyl butyrate | ester | 0.27 | - | - | - | - | - | - | - | - | - | - | - |
| 14.63 | (*E*,*E*)-2,4-Heptadienal | aldehyde | 353.07 | 1439.87 | 994.00 | 952.65 | 726.89 | 694.50 | 1229.84 | 610.82 | 736.54 | 472.34 | 412.06 | 70.02 |
| 14.68 | Decane | hydrocarbon | 0.36 | - | - | - | - | - | - | - | - | - | - | - |
| 14.86 | Octanal | aldehyde | 1.08 | 0.58 | 0.43 | 0.32 | 0.47 | 0.47 | 0.45 | 0.32 | 0.45 | 0.32 | 0.33 | 0.44 |
| 16.17 | 5-Ethylcyclopent-1-enecarboxaldehyde | aldehyde | - | - | 0.67 | - | 0.42 | 0.44 | 0.83 | 0.32 | - | - | - | - |
| 16.32 | 2-Ethyl-1-Hexanol | alcohol | 0.33 | 1.98 | 1.75 | 1.48 | 1.28 | 1.21 | 2.40 | 1.78 | 2.01 | 1.49 | 1.21 | 0.73 |
| 16.46 | 2,2,6-trimethyl-Cyclohexanone | ketone | 0.68 | - | 0.50 | 0.32 | - | - | 0.71 | 0.40 | - | 0.31 | - | - |
| 16.70 | Benzyl alcohol | alcohol | 2.99 | 2.12 | 2.75 | 3.13 | 3.01 | 3.31 | 2.36 | 3.38 | 3.70 | 3.76 | 4.08 | 3.25 |
| 16.85 | 3-Octen-2-one | ketone | 1.37 | 6.38 | 4.66 | 3.34 | 3.41 | 3.15 | 6.89 | 3.42 | 2.89 | 1.79 | 0.86 | - |
| 17.13 | Benzeneacetaldehyde | aldehyde | 2661.32 | 4363.52 | 2958.70 | 3111.10 | 2221.11 | 2103.27 | 3400.57 | 2010.51 | 2174.66 | 1500.85 | 1805.53 | 739.14 |
| 17.55 | 5-Ethyldihydro-2(3H)-furanone | ester | 1.36 | 0.69 | 0.81 | - | 0.90 | 1.01 | - | - | - | 1.23 | 1.13 | 1.03 |
| 17.75 | 2,6-Dimethylnonane | hydrocarbon | 0.37 | - | - | - | - | - | - | - | - | - | - | - |
| 17.92 | (*E*)-2-Octenal | aldehyde | 4.42 | 15.65 | 10.09 | 5.16 | 2.07 | 4.11 | 9.63 | 3.65 | 2.19 | 0.00 | 0.00 | 0.00 |
| 18.15 | Acetophenone | ketone | 2.64 | 7.42 | 4.40 | 3.58 | 4.57 | 3.00 | 9.17 | 9.48 | 0.00 | 0.00 | 0.00 | 0.00 |
| 18.35 | 2-Methyl-benzaldehyde, | aldehyde | - | 0.39 | 0.31 | 0.24 | 0.20 | 0.20 | 0.38 | 0.19 | 0.20 | - | - | - |
| 18.78 | 1-Octanol | alcohol | 1.39 | 7.69 | 5.59 | 4.74 | 4.47 | 4.46 | 6.59 | 5.48 | 6.46 | 5.86 | 5.10 | 2.43 |
| 19.52 | *Trans*-linalool oxide (furanoid) | alcohol | 1.21 | 5.19 | 4.52 | 4.63 | 3.42 | 3.79 | 6.11 | 5.34 | 6.35 | 5.74 | 5.61 | 3.16 |
| 19.93 | 3,5-Octadien-2-one | ketone | 155.96 | 714.73 | 508.87 | 431.61 | 369.87 | 394.04 | 762.90 | 544.06 | 259.90 | 104.22 | 154.61 | 121.18 |
| 20.58 | Linalool | alcohol | 11.36 | 112.93 | 70.23 | 63.29 | 48.74 | 50.44 | 90.04 | 73.24 | 88.25 | 80.80 | 66.93 | 25.39 |
| 20.60 | 1-Nonanal | aldehyde | 3.65 | - | - | - | - | - | - | - | - | - | - | - |
| 20.82 | Dihydrolinalool | alcohol | 11.25 | 59.52 | 44.50 | 42.36 | 34.99 | 35.29 | 58.55 | 49.10 | 53.37 | 52.15 | 48.86 | 23.27 |
| 21.08 | (3*E*)-4,8-dimethylnona-1,3,7-triene | hydrocarbon | 1.35 | - | - | - | - | - | - | - | - | - | - | - |
| 21.10 | Phenylethyl alcohol | alcohol | 23.03 | 15.36 | 25.28 | 27.87 | 25.29 | 27.96 | 22.71 | 29.50 | 31.73 | 31.17 | 33.50 | 26.10 |
| 21.96 | *p*-Mentha-1,5,8-triene | hydrocarbon | 0.26 | - | 0.43 | 0.40 | 0.32 | 0.35 | - | - | - | - | - | - |
| 22.70 | Benzyl nitrile | other | 30.15 | 78.97 | 69.97 | 61.75 | 56.62 | 58.19 | 81.38 | 84.55 | 89.27 | 81.88 | 78.67 | 54.48 |
| 22.71 | 5-ethyl-6-methyl-3-hepten-2-one | ketone | 0.57 | 1.25 | 1.05 | 0.71 | 0.61 | 0.59 | 0.85 | 0.57 | - | - | 0.64 | - |
| 22.72 | *Trans*-3-nonen-2-one | ketone | 0.26 | 3.95 | 3.12 | 1.90 | 2.00 | 1.75 | 2.52 | 0.82 | 0.68 | 0.39 | 0.53 | 0.00 |
| 23.33 | Atropaldehyde | aldehyde | - | - | - | - | - | - | - | - | 0.88 | 0.41 | - | 1.27 |
| 23.86 | 1-Phenyl-1-propanone | ketone | 0.11 | 0.30 | 0.27 | 0.22 | - | 0.13 | 0.25 | 0.25 | 0.25 | 0.26 | 0.19 | 0.09 |
| 24.25 | 1-(4-Methylphenyl)-ethanone | ketone | - | 0.11 | - | - | - | - | - | - | - | - | - | - |
| 24.29 | (*E*)-linalool oxide (pyranoid) | alcohol | 3.79 | 3.96 | 6.48 | 7.90 | 6.82 | 8.05 | 5.58 | 6.34 | 7.96 | 8.21 | 8.45 | 5.96 |
| 25.52 | Methyl salicylate | ester | 8.11 | 55.58 | 41.25 | 28.78 | 28.61 | 26.91 | 41.22 | 31.14 | 30.62 | 22.64 | 16.87 | 5.22 |
| 25.58 | 2,6-Dimethyl-3,7-octadiene-2,6-diol | alcohol | 0.77 | - | - | - | - | - | - | - | - | - | - | - |
| 25.80 | *α*-Terpineol | alcohol | - | 2.79 | 2.36 | 1.59 | 1.59 | 1.55 | 2.24 | 1.90 | 2.81 | 2.83 | 2.92 | 1.72 |
| 25.95 | Safranal | aldehyde | 0.45 | 1.86 | 1.62 | 1.27 | 1.02 | 1.10 | 1.51 | 1.45 | 1.69 | 1.28 | 0.86 | 0.43 |
| 26.24 | Dodecane | hydrocarbon | 0.72 | - | - | - | - | - | - | - | - | - | - | - |
| 26.39 | 2-Propyl-phenol | phenol | 6.34 | 36.33 | 25.93 | 22.15 | 16.93 | 18.25 | 31.63 | 22.76 | 27.28 | 24.08 | 30.47 | 18.07 |
| 26.65 | Decanal | aldehyde | 1.71 | 2.23 | 2.06 | 2.19 | 2.14 | 2.02 | 2.10 | 1.78 | 3.44 | 2.83 | 2.90 | 3.19 |
| 26.88 | 3,4-Dimethyl-benzaldehyde | aldehyde | 0.55 | 0.09 | 1.77 | 4.33 | 1.34 | 0.94 | 2.14 | 1.13 | 1.10 | 1.12 | 0.93 | 0.49 |
| 26.94 | 2-Amino-benzaldehyde | aldehyde | 0.14 | 0.09 | - | - | - | - | - | - | - | - | - | - |
| 26.94 | N-phenyl-formamide | other | - | - | - | - | 0.06 | - | - | - | - | - | - | 0.08 |
| 27.25 | *β*-Cyclocitral | aldehyde | 3.20 | 6.41 | 6.14 | 3.69 | 3.41 | 3.25 | 6.17 | 4.27 | 4.63 | 4.55 | 3.72 | 1.68 |
| 27.56 | 1-Phenyl-2-butanone | ketone | - | 0.48 | 0.28 | 0.24 | 0.35 | 0.21 | 0.43 | 0.34 | 0.35 | 0.28 | 0.27 | 0.14 |
| 27.72 | (*Z*)-2,6-Octadien-1-ol, 3,7-dimethy | alcohol | - | 0.99 | 0.82 | - | 0.47 | - | 0.81 | 0.78 | 1.00 | 1.08 | 1.17 | 0.78 |
| 28.67 | iso-Geraniol | alcohol | - | - | - | - | - | - | - | 0.65 | 0.98 | 0.95 | 0.90 | 1.16 |
| 29.41 | 2-Phenylethyl ester-acetic acid | ester | 0.78 | 6.14 | 4.46 | 4.60 | 4.87 | 3.41 | 7.12 | 4.69 | 6.54 | 5.73 | 5.70 | 3.35 |
| 29.44 | Geraniol | alcohol | 51.54 | 474.25 | 351.85 | 376.52 | 291.19 | 343.49 | 402.70 | 342.78 | 456.00 | 476.06 | 549.28 | 400.53 |
| 30.06 | 2-Phenylbut-2-enal | aldehyde | - | - | - | - | - | - | - | 0.52 | - | - | - | - |
| 31.17 | *γ*-Octalactone | ester | - | - | - | - | - | - | - | - | 0.03 | 0.03 | - | - |
| 32.29 | 2H-pyran-2-one, tetrahydro-6-propyl | ester | - | - | - | - | - | 2.41 | - | - | - | - | - | - |
| 32.34 | Indole | heterocyclic compound | 12617.13 | 21655.94 | 21490.84 | 21251.59 | 17919.99 | 19913.35 | 22021.99 | 18188.20 | 18449.12 | 18527.24 | 25150.76 | 20792.51 |
| 32.35 | (*E*,*E*)-2,4-Decadienal | aldehyde | 0.71 | 1.78 | 1.26 | 1.36 | 1.18 | 1.60 | 0.94 | 0.93 | 2.24 | 2.60 | 2.35 | 1.87 |
| 32.59 | (2-Nitroethyl)-benzene | other | 30.59 | 87.01 | 75.93 | 73.67 | 68.53 | 68.05 | 73.15 | 69.38 | 71.26 | 74.39 | 79.70 | 66.52 |
| 32.95 | 4-(1-Methylpropyl)-phenol | phenol | - | 1.03 | 0.64 | 0.52 | - | 0.41 | - | - | 0.46 | - | 0.35 | 0.15 |
| 33.51 | 4,6-Dimethyldodecane | hydrocarbon | 0.98 | - | - | - | - | - | - | - | - | - | - | - |
| 33.64 | Methyl 2-methoxybenzoate | ester | - | - | - | - | - | - | - | - | - | - | 0.23 | - |
| 33.86 | Methyl anthranilate | ester | 0.46 | 0.56 | 0.42 | 0.39 | 0.26 | 0.53 | 0.21 | 0.28 | 0.36 | 0.24 | 0.33 | 0.43 |
| 34.02 | 5-Ethyl-1,2,3,4-tetrahydro-naphthalene | hydrocarbon | - | - | 1.31 | - | - | 1.04 | 1.13 | 0.61 | 0.98 | 0.93 | 0.99 | 0.66 |
| 34.20 | Butanoic acid, phenylmethyl ester | ester | - | 0.52 | - | - | - | - | - | - | - | - | - | - |
| 34.23 | Dihydro-5-pentyl-2(3H)-Furanone | ester | - | 0.75 | 0.55 | 0.38 | 0.51 | 0.49 | 0.50 | 0.37 | 0.52 | 0.41 | 0.43 | 0.61 |
| 34.85 | *γ*-Nonanolactone | ester | - | - | 2.45 | 2.41 | 1.63 | 2.50 | 2.03 | 1.75 | 2.21 | 2.08 | 2.18 | 1.96 |
| 35.34 | Teksanol | ester | - | 0.54 | 0.62 | 0.61 | 0.64 | 0.49 | 1.24 | - | 0.86 | 0.44 | 0.51 | 0.72 |
| 35.85 | Hexenyl hexanoate | ester | 3.96 | 3.26 | - | - | - | - | - | - | - | 1.01 | 0.96 | - |
| 36.64 | Jasmone | ketone | 250.14 | 1120.18 | 1387.19 | 1028.74 | 942.01 | 966.17 | 1300.00 | 1232.50 | 1275.27 | 1415.19 | 1410.40 | 1443.78 |
| 36.84 | Tetradecane | hydrocarbon | 0.63 | - | - | - | - | - | - | - | - | - | - | - |
| 36.97 | *Trans*-isoeugenol | phenol | 2.81 | 5.47 | 5.61 | 5.87 | 5.68 | 6.72 | 2.95 | 2.37 | 2.85 | 5.39 | 2.49 | 2.57 |
| 37.81 | *α*-Ionone | ketone | 2.06 | 10.73 | 8.66 | 7.42 | 6.46 | 7.23 | 8.74 | 6.60 | 9.51 | 8.89 | 10.96 | 6.78 |
| 38.17 | Coumarin | ester | 0.12 | - | 0.04 | - | - | - | 0.04 | - | - | - | - | - |
| 39.00 | 6,10-dimethyl-5,9-Undecadien-2-one | ketone | - | 5.36 | 3.17 | 2.01 | 2.22 | 3.20 | 3.04 | 2.02 | 3.93 | 3.94 | 3.87 | 2.81 |
| 39.19 | (*E*)-*β*-farnesene | hydrocarbon | 3.93 | 6.83 | 7.10 | 4.30 | 4.91 | 4.96 | 4.24 | 4.55 | 6.89 | 6.69 | 7.85 | 6.55 |
| 39.63 | 5-Hexyldihydro-2(3H)-furanone | ester | - | 1.10 | 0.98 | 0.94 | 0.86 | 1.07 | 1.13 | 0.82 | 1.20 | 1.32 | 1.52 | 1.75 |
| 40.12 | *β*-Ionone | ketone | 5.81 | 51.44 | 35.11 | 25.12 | 25.85 | 26.66 | 35.87 | 27.68 | 38.21 | 41.07 | 44.91 | 31.30 |
| 40.27 | *β*-ionone 5,6-epoxide | ketone | 2.04 | 10.74 | 10.43 | 9.33 | 8.14 | 9.35 | 12.00 | 9.05 | 11.72 | 11.83 | 13.95 | 11.33 |
| 40.40 | 2-Phenylethyl-2-methylbutyrate | ester | 2.86 | 12.46 | 7.59 | 4.35 | 5.17 | 6.23 | 7.13 | 5.74 | 9.53 | 9.76 | 9.84 | 6.60 |
| 40.54 | Jasmine lactone | ester | 18644.50 | 6562.01 | 9018.92 | 12652.92 | 14535.58 | 17980.80 | 6300.69 | 4606.95 | 4417.10 | 4429.21 | 4800.52 | 4253.31 |
| 40.59 | 5-Decanolide | ester | - | 5.18 | 6.45 | 7.63 | 5.06 | 7.28 | 5.53 | 3.79 | 4.43 | 4.47 | 6.73 | 3.99 |
| 40.95 | Butylated Hydroxytoluene | alcohol | 0.14 | 0.13 | - | - | - | - | - | - | - | - | - | - |
| 41.06 | *α*-Farnesene | hydrocarbon | 35.57 | 3.04 | 2.63 | 1.55 | 1.50 | 1.82 | 1.32 | 1.42 | 2.18 | 2.28 | 2.71 | 2.25 |
| 41.12 | *β*-Bisabolene | hydrocarbon | - | 0.92 | 0.80 | 0.43 | 0.38 | 0.48 | 0.43 | 0.46 | 0.75 | 0.75 | 0.94 | 0.76 |
| 41.33 | 2,4-Di-tert-butylphenol | phenol | - | 1.86 | 1.63 | 2.28 | 2.17 | 1.87 | 3.15 | 2.84 | 3.29 | 2.32 | - | - |
| 41.65 | Dihydroactinidiolide | ester | 0.90 | - | - | 0.54 | 0.51 | 0.55 | - | - | - | - | - | - |
| 42.08 | *α*-Calacorene | hydrocarbon | - | - | - | - | 0.09 | 0.10 | - | - | - | - | - | - |
| 42.13 | (*E*)-*α*-Bisabolene | hydrocarbon | - | - | 0.54 | - | - | - | - | - | 0.40 | 0.43 | - | 0.32 |
| 42.62 | 3,7-Dimethyl-6-Octene-1,2,3-triol | alcohol | 2.46 | 7.52 | 7.98 | 7.91 | 6.57 | 6.94 | 10.29 | 8.25 | 9.46 | 9.61 | 10.32 | 9.38 |
| 42.74 | *Trans*-nerolidol | alcohol | 709.23 | 1284.91 | 929.64 | 569.67 | 722.71 | 772.89 | 849.67 | 835.98 | 1224.73 | 1479.61 | 1780.89 | 1567.75 |
| 43.23 | Hexyl benzoate | ester | 0.35 | - | - | - | - | - | - | - | - | - | - | - |
| 43.45 | 5,9-Undecatrien-2-one | ketone | - | - | - | - | 0.43 | - | - | - | - | - | - | 0.28 |
| 43.54 | Diethyl Phthalate | ester | - | 0.98 | 1.09 | 1.22 | 2.03 | 1.29 | 1.02 | 0.63 | 0.78 | 0.66 | 0.54 | 0.49 |
| 43.86 | Hexadecane | hydrocarbon | 0.61 | - | - | - | - | - | - | - | - | - | - | - |
| 44.04 | (-)-Epicedrol | alcohol | - | - | - | - | - | 0.64 | - | - | - | - | - | - |
| 44.11 | Cedrol | alcohol | 0.36 | 1.00 | 0.88 | 0.63 | 0.89 | - | 1.52 | 0.74 | 1.34 | 0.78 | 0.82 | 1.80 |
| 44.49 | Benzophenone | ketone | 0.23 | 2.79 | 1.92 | 1.61 | 3.99 | 2.09 | 1.40 | 1.25 | 1.65 | 1.42 | 1.68 | 1.31 |
| 44.53 | Cadinenol | other | - | - | - | - | - | - | - | - | - | - | 0.55 | 0.45 |
| 44.78 | 2-Phenethyl hexanoate | ester | 1.93 | - | - | - | - | - | - | - | - | - | - | - |
| 44.86 | Methyl jasmonate | ester | 326.05 | 370.29 | 633.49 | 737.50 | 693.74 | 735.74 | 762.17 | 545.34 | 282.74 | 664.58 | 656.97 | 69.52 |
| 45.17 | *α*-Cadinol | alcohol | - | 0.88 | 0.82 | 0.74 | 0.66 | 0.90 | 0.86 | 0.60 | 0.95 | 0.89 | 1.20 | 1.15 |
| 45.80 | Hexanedioic acid, bis(2-methylpropyl) ester | ester | - | 2.32 | 2.58 | 2.68 | 2.19 | 2.50 | - | - | - | - | - | - |
| 46.49 | 2,6,10-Dodecatrien-1-ol-3,7,11-trimethyl | alcohol | 1.19 | 2.24 | 1.47 | 1.14 | 1.41 | 1.80 | 1.48 | 1.23 | 2.45 | 2.22 | 3.06 | 2.44 |
| 47.29 | 3,5-Di-tert-butyl-4-hydroxybenzaldehyde | aldehyde | - | 0.11 | 0.09 | 0.10 | 0.14 | 0.11 | - | - | 0.07 | 0.14 | 0.06 | 0.08 |
| 47.53 | Benzyl Benzoate | ester | - | 0.22 | 0.14 | 0.09 | 0.11 | 0.16 | - | 0.09 | 0.18 | 0.16 | 0.21 | 0.16 |
| 48.03 | 1,1'-Biphenyl, 2,2'-diethyl- | alcohol | - | - | - | - | 0.14 | - | - | - | - | - | - | - |
| 48.81 | 4,8,12-Tetradecatrienal-5,9,13-trimethyl | aldehyde | - | 0.48 | 0.39 | 0.37 | - | 0.37 | - | - | 0.59 | 0.46 | 0.58 | 0.32 |
| 49.24 | 2-Phenylethyl ester-benzoic acid | ester | - | 0.23 | 0.14 | 0.09 | 0.11 | 0.15 | - | - | 0.19 | 0.20 | 0.22 | 0.18 |
| 49.29 | Diisobutyl phthalate | ester | - | 1.12 | 1.22 | 1.15 | 0.98 | 1.09 | 0.49 | 0.29 | 0.42 | 0.54 | 0.39 | 0.49 |
| 50.14 | Dibutyl phthalate | ester | - | 1.20 | 1.24 | 1.28 | 1.11 | 1.19 | 0.88 | 0.63 | 0.92 | 0.67 | 0.70 | 0.52 |
| 53.27 | 11,14-Octadecadienoic acid methyl ester | ester | 0.25 | - | - | - | - | - | - | - | - | - | - | - |
| 53.36 | Methyl linolenate | ester | 0.44 | - | - | - | - | - | - | - | - | - | - | - |

JGY represented the JGY tea; 40, 50, 60, 70, and 80 ℃ represented the tea hydrolats were collected under 50, 60, 70, and 80 ℃, respectively; 0-10, 10-20, 20-40, 40-80, 80-160, 160-240 represented the hydrolats were collected during 0-10, 10-20, 20-40, 40-80, 80-160, 160-240 min periods, respectively. RT: Retention indices were determined using a homologous series of *n*-alkanes (C_7_-C_40_).

**Table S4.** The quantitative results of key active components in 50 ℃, 40 min tea hydrolat sample.

| Components | Concentration (μg/L) | Linear equation | R^2^ | Range (μg/L) |
| --- | --- | --- | --- | --- |
| 1-Penten-3-one | 75.70 | y = 42.705x + 0.0516 | 0.9903 | 2-100 |
| Hexanal | 86.35 | y = 67.462x + 0.1398 | 0.9988 | 5-500 |
| (*Z*)-3-hexen-1-ol | 420.42 | y = 487.18x + 0.027 | 0.9999 | 25-1000 |
| *Cis*-4-heptenal | 1.37 | y = 112.11x - 0.0301 | 0.9971 | 1-10 |
| Benzaldehyde | 186.36 | y = 11.607x - 0.2135 | 0.9935 | 10-300 |
| Methyl heptenone | 500.56 | y = 24.785x - 1.8491 | 0.9993 | 10-1000 |
| (*E*,*E*)-2,4 heptadienal | 970.86 | y = 29.699x - 2.6732 | 0.9998 | 50-2500 |
| Benzeneacetaldehyde | 2792.01 | y = 118.32x - 8.7834 | 0.9960 | 50-7500 |
| (*E*)-2-octenal | 6.52 | y = 4.396x - 0.1058 | 0.9924 | 2-20 |
| Acetophenone | 7.16 | y = 11.287x - 0.0581 | 0.9980 | 2-20 |
| 3,5-Octadien-2-one | 596.62 | y = 17.799x - 0.2463 | 0.9997 | 25-1000 |
| Linalool | 85.63 | y = 1.6074x - 0.3694 | 0.9993 | 10-200 |
| 3-Nonen-2-one | 1.70 | y = 3.8412x - 0.059 | 0.9879 | 1-10 |
| Methyl salicylate | 36.42 | y = 5.2951x - 0.2166 | 0.9998 | 10-80 |
| 2-Propylphenol | 28.54 | y = 13.257x - 0.0187 | 0.9988 | 2-20 |
| Geraniol | 400.52 | y = 10.632x - 0.1905 | 0.9997 | 25-1000 |
| Indole | 20297.00 | y = 47.565x + 16.255 | 0.9985 | 800-40000 |
| Jasmone | 1278.08 | y = 12.236x - 0.4718 | 0.9997 | 50-2000 |
| *β*-Ionone | 34.46 | y = 1.6996x - 0.2292 | 0.9931 | 10-80 |
| Jasmine lactone | 5470.10 | y = 372.25x - 20.363 | 0.9996 | 2000-20000 |
| *Trans*-nerolidol | 931.42 | y = 4.8201x - 13.364 | 0.9920 | 100-1600 |
| Methyl jasmonate | 601.72 | y = 136.13x + 0.4125 | 0.9977 | 25-1000 |

**Table S5.** Changes in the content of volatiles under different scenting treatments.

| Compounds | Category | Content (μg/L) | | | | | | | | | | | |
| --- | --- | --- | --- | --- | --- | --- | --- | --- | --- | --- | --- | --- | --- |
|  |  | 4h residues | | | | 40 min residues | | | | JGY | | | |
|  |  | ck1 | 9:1 | 6:1 | 3:1 | ck2 | 9:1 | 6:1 | 3:1 | ck3 | 9:1 | 6:1 | 3:1 |
| *Trans*-nerolidol | Alcohol (18) | 397.02 | 454.54 | 436.48 | 488.65 | 470.33 | 498.79 | 513.93 | 530.97 | 473.59 | 558.76 | 724.65 | 752.69 |
| (*Z*)-3-Hexen-1-ol |  | 323.47 | 209.11 | 228.36 | 240.26 | 251.36 | 199.19 | 282.85 | 305.87 | 225.95 | 251.55 | 256.82 | 219.69 |
| Geraniol |  | 24.15 | 24.79 | 23.73 | 25.19 | 20.85 | 26.09 | 26.06 | 27.80 | 38.65 | 48.40 | 47.36 | 48.22 |
| Phenylethyl alcohol |  | 12.29 | 13.21 | 12.57 | 11.85 | 10.96 | 11.83 | 12.27 | 12.18 | 15.77 | 17.14 | 16.96 | 16.83 |
| Linalool |  | 3.80 | 4.73 | 4.26 | 4.14 | 5.13 | 6.17 | 5.44 | 6.31 | 9.42 | 11.44 | 9.44 | 8.99 |
| Dihydrolinalool |  | 2.76 | 4.00 | 3.60 | 3.42 | 5.13 | 5.60 | 4.46 | 4.78 | 8.95 | 10.74 | 9.16 | 8.82 |
| 3,7-Dimethyl-6-octene-1,2,3-triol |  | 2.07 | 1.68 | 2.47 | 2.67 | - | - | - | - | - | - | - | - |
| *α*-Terpineol |  | 1.32 | 0.26 | 0.26 | 0.29 | - | - | - | - | 0.36 | 0.56 | 0.65 | 0.59 |
| (*E*)-linalool oxide (pyranoid) |  | 1.93 | 2.10 | 2.04 | 1.94 | 1.66 | 1.84 | 1.77 | 1.86 | 2.39 | 2.66 | 2.48 | 2.58 |
| Benzyl alcohol |  | 1.13 | 1.22 | 1.12 | 1.05 | 0.84 | 1.03 | 1.02 | 1.03 | 1.43 | 1.55 | 1.55 | 1.47 |
| 1-Octen-3-ol |  | 0.41 | 0.47 | 0.58 | 0.59 | 0.58 | 0.58 | 0.61 | 0.56 | 1.39 | 1.00 | 0.89 | 0.69 |
| 1-Octanol |  | 0.47 | 0.48 | 0.65 | 0.57 | 0.64 | 0.65 | 0.76 | 0.66 | 0.81 | 1.13 | 1.22 | 1.07 |
| 1-Pentanol |  | 0.18 | 0.21 | 0.21 | 0.23 | 0.16 | 0.22 | 0.25 | 0.22 | 0.35 | 0.30 | 0.30 | 0.18 |
| Butylated Hydroxytoluene |  | 0.25 | 0.29 | 0.14 | 0.15 | 0.19 | - | 0.19 | 0.15 | 0.22 | 0.14 | 0.15 | 0.25 |
| Cedrol |  | 0.22 | 0.27 | 0.22 | 0.28 | 0.22 | 0.28 | 0.25 | 0.25 | 0.27 | 0.25 | 0.24 | 0.24 |
| 1-Heptanol |  | - | - | - | - | - | - | - | - | 0.16 | 0.23 | 0.22 | 0.19 |
| 2-Ethyl-1-hexanol |  | - | - | - | - | - | - | - | - | 0.27 | 0.34 | 0.39 | 0.48 |
| *Trans*-linalool oxide (furanoid) |  | - | - | - | - | - | - | - | - | 0.76 | 0.86 | 0.66 | 0.70 |
| Benzeneacetaldehyde | Aldehyde (17) | 1762.02 | 2225.87 | 2154.94 | 2078.73 | 1602.02 | 1523.38 | 1718.94 | 1808.61 | 1953.23 | 2041.17 | 1758.11 | 1906.89 |
| Hexanal |  | 56.04 | 48.40 | 62.91 | 46.59 | 71.22 | 52.47 | 73.81 | 72.95 | 163.98 | 145.21 | 132.81 | 87.45 |
| Benzaldehyde |  | 32.77 | 39.56 | 42.37 | 44.06 | 38.57 | 46.99 | 45.51 | 47.69 | 103.39 | 102.61 | 97.66 | 74.81 |
| 1-Nonanal |  | 4.59 | 4.10 | 6.59 | 5.57 | 5.06 | 4.26 | 4.86 | 3.61 | 3.57 | 4.31 | 4.58 | 4.22 |
| *β*-Cyclocitral |  | 2.33 | 3.04 | 3.10 | 3.09 | 3.39 | 3.54 | 3.63 | 3.53 | 4.47 | 5.45 | 6.12 | 5.44 |
| Decanal |  | 2.36 | 1.62 | 3.13 | 3.39 | 2.07 | 1.73 | 1.72 | 1.44 | 0.83 | 1.25 | 1.28 | 1.19 |
| *Cis*-4-Heptenal |  | 1.53 | 1.95 | 2.20 | 1.20 | 2.18 | 1.86 | 1.89 | 2.05 | 4.86 | 3.21 | 2.38 | 1.92 |
| (*E*,*E*)-2,4-Heptadienal |  | 1.37 | 1.27 | 1.66 | 1.23 | 16.46 | 16.17 | 25.60 | 17.68 | 283.39 | 274.65 | 298.68 | 185.56 |
| Octanal |  | 0.81 | 0.86 | 1.33 | 1.14 | 1.05 | 0.84 | 0.95 | 0.77 | 1.34 | 1.13 | 1.12 | 0.93 |
| (*E*)-2-Octenal |  | 0.55 | 0.50 | 0.76 | 1.05 | 1.41 | 1.24 | 1.08 | 1.48 | 4.98 | 3.49 | 4.28 | 2.52 |
| Heptaldehyde |  | 0.65 | 0.62 | 0.76 | 0.53 | 0.71 | 0.60 | 0.65 | 0.61 | 0.90 | 0.89 | 0.80 | 0.64 |
| Pentanal |  | 0.52 | 0.44 | 0.59 | 0.52 | 0.47 | 0.42 | 0.50 | 0.42 | 0.59 | 0.51 | 0.36 | 0.29 |
| (*E*,*E*)-2,4-Decadienal |  | 0.38 | 0.33 | 0.29 | 0.34 | - | - | - | - | - | - | - | - |
| Safranal |  | 0.21 | 0.42 | 0.39 | 0.40 | 0.42 | 0.50 | 0.47 | 0.50 | 0.63 | 0.81 | 0.81 | 0.72 |
| (*E*)-2-Pentenal |  | 0.12 | 0.12 | 0.12 | 0.09 | 0.18 | 0.14 | 0.17 | - | 0.45 | 0.32 | 0.29 | 0.23 |
| 2-Phenylbut-2-enal |  | 0.07 | 0.08 | 0.08 | 0.08 | 0.09 | 0.10 | 0.10 | 0.12 | 0.15 | 0.18 | 0.19 | 0.14 |
| 2-Amino-benzaldehyde |  | - | - | - | - | - | - | - | - | 0.13 | 0.16 | 0.15 | 0.12 |
| Jasmone | Ketone (14) | 156.74 | 172.06 | 165.72 | 180.80 | 154.19 | 183.60 | 164.63 | 188.70 | 220.19 | 270.20 | 268.18 | 277.26 |
| 3,5-Octadien-2-one |  | 10.25 | 13.80 | 17.63 | 19.13 | 36.68 | 47.85 | 46.22 | 47.88 | 162.95 | 157.57 | 163.14 | 126.93 |
| *β*-ionone |  | 9.13 | 10.51 | 10.72 | 10.49 | 10.08 | 11.30 | 10.32 | 12.37 | 12.92 | 16.11 | 20.28 | 19.38 |
| Methyl heptenone |  | 7.51 | 28.72 | 29.93 | 17.53 | 59.30 | 64.57 | 55.21 | 58.47 | 198.84 | 203.19 | 187.82 | 141.28 |
| 1-Penten-3-one |  | 7.65 | 8.49 | 8.56 | 7.38 | 11.30 | 13.25 | 14.22 | 13.26 | 14.70 | 11.88 | 20.97 | 8.47 |
| *α*-Ionone |  | 1.47 | 1.59 | 1.82 | 1.48 | 1.62 | 1.99 | 1.58 | 1.97 | 1.75 | 2.08 | 2.49 | 2.49 |
| 6,10-Dimethyl-5,9-undecadien-2-one |  | 1.14 | 1.19 | 1.26 | 1.38 | 1.26 | 1.25 | 1.35 | 1.34 | 2.26 | 1.75 | 2.13 | 1.86 |
| 2,2,6-Trimethyl-cyclohexanone |  | 0.50 | 0.60 | 0.61 | 0.50 | 0.69 | 0.68 | 0.72 | 0.68 | 0.81 | 0.98 | 0.91 | 0.83 |
| *Trans*-3-nonen-2-one |  | 0.22 | 0.26 | 0.29 | 0.35 | 0.39 | 0.37 | 0.49 | 0.40 | 0.54 | 0.78 | 0.92 | 0.75 |
| Acetophenone |  | 0.15 | 0.08 | 0.07 | 0.13 | 0.07 | 0.09 | 0.09 | 0.10 | 0.71 | 0.66 | 0.77 | 0.48 |
| 3-Octen-2-one |  | - | - | - | - | 0.26 | 0.31 | 0.36 | 0.29 | 1.14 | 1.19 | 1.21 | 0.74 |
| 1-Phenyl-1-propanone, |  | - | - | - | - | - | - | - | - | 0.05 | 0.06 | 0.04 | 0.04 |
| *β*-ionone-5,6-epoxide |  | - | - | - | - | 2.12 | 2.48 | 2.79 | 2.67 | 2.84 | 3.29 | 3.68 | 3.42 |
| Benzophenone |  | - | - | - | - | 0.22 | 0.23 | 0.30 | 0.26 | 0.15 | 0.19 | 0.21 | - |
| Jasminlactone | Ester (12) | 10049.88 | 12123.87 | 13373.47 | 12641.79 | 11350.98 | 13753.43 | 13890.17 | 12996.10 | 11504.86 | 14325.83 | 14407.89 | 15680.32 |
| Methyl jasmonate |  | 201.84 | 226.20 | 245.56 | 240.83 | 197.15 | 205.42 | 227.56 | 247.45 | 168.15 | 307.92 | 291.60 | 310.27 |
| 2-Phenylethyl-2-methylbutyrate |  | 1.57 | 2.18 | 1.95 | 2.38 | 2.35 | 2.24 | 2.25 | 2.36 | 2.79 | 3.32 | 3.99 | 3.54 |
| 2-Phenethyl hexanoate |  | 1.28 | 1.50 | 1.21 | 1.54 | 1.44 | 1.53 | 1.49 | 1.53 | 1.60 | 1.88 | 2.35 | 2.26 |
| Hexenyl hexanoate |  | 1.17 | 1.60 | 1.41 | 1.46 | 1.98 | 1.85 | 1.83 | 1.80 | 2.45 | 3.48 | 4.54 | 4.02 |
| Dihydroactinidiolide |  | 1.03 | 1.28 | 1.12 | 1.20 | 1.08 | 1.21 | 1.20 | 1.20 | 1.15 | 1.44 | 1.69 | 1.83 |
| Methyl salicylate |  | 0.61 | 0.72 | 0.60 | 0.57 | 0.75 | 1.04 | 0.96 | 0.78 | 7.66 | 9.19 | 9.08 | 8.31 |
| 5-Ethyldihydro-2(3H)-furanone |  | 0.54 | 0.65 | 0.55 | 0.46 | 0.50 | 0.52 | 0.51 | 0.59 | 0.67 | 0.72 | 0.68 | 0.69 |
| Methyl linolenate |  | 0.19 | 0.31 | 0.36 | 0.58 | 0.35 | 0.49 | 0.42 | 0.36 | 0.31 | 0.36 | 0.60 | 0.62 |
| Dibutyl phthalate |  | 0.10 | 0.14 | 0.11 | 0.15 | 0.10 | 0.14 | 0.10 | 0.16 | 0.12 | 0.19 | 0.21 | 0.17 |
| Methyl anthranilate |  | - | - | - | - | 0.06 | 0.09 | - | - | 0.10 | 0.15 | 0.01 | 0.20 |
| Hexyl benzoate |  | - | - | - | - | 0.25 | - | 0.27 | 0.27 | 0.33 | 0.33 | 0.51 | 0.45 |
| *α*-Farnesene | Hydrocarbon (13) | 15.14 | 23.00 | 19.27 | 23.70 | 21.89 | 19.34 | 22.64 | 21.68 | 17.43 | 18.83 | 24.02 | 28.55 |
| (*E*)-*β*-farnesene |  | 5.14 | 7.32 | 5.99 | 6.40 | 5.95 | 5.70 | 5.64 | 5.42 | 5.11 | 5.65 | 6.86 | 6.44 |
| 2,6-Dimethylnonane |  | 0.85 | 0.76 | 1.33 | 1.07 | 1.30 | 0.95 | 0.94 | 1.40 | 1.20 | 1.22 | 1.87 | 1.20 |
| Styrene |  | 0.47 | 1.53 | 0.54 | 0.48 | 2.59 | 1.82 | 1.92 | 2.43 | 3.48 | 1.15 | 1.07 | 0.65 |
| Tetradecane |  | 0.41 | 0.40 | 0.43 | 0.37 | 0.30 | 0.35 | 0.36 | 0.31 | 0.38 | 0.31 | 0.41 | 0.39 |
| (*E*)-*α*-Bisabolene |  | 0.32 | 0.48 | 0.38 | 0.44 | 0.37 | 0.36 | 0.38 | 0.35 | 0.31 | 0.36 | 0.47 | 0.43 |
| Dodecane |  | 0.42 | 0.44 | 0.47 | 0.38 | 0.38 | 0.45 | 0.49 | 0.42 | 0.38 | 0.42 | 0.53 | 0.49 |
| 4,6-Dimethyldodecane |  | 0.43 | 0.51 | 0.59 | 0.43 | 0.79 | - | 0.56 | 0.74 | 0.65 | 0.70 | 0.57 | 0.75 |
| Decane |  | 0.23 | 0.27 | 0.34 | 0.24 | - | - | - | - | - | - | - | - |
| p-Mentha-1,5,8-triene |  | 0.12 | 0.15 | 0.13 | 0.12 | - | - | - | - | 0.29 | 0.35 | 0.37 | 0.21 |
| Calamenene |  | 0.11 | 0.13 | 0.11 | 0.48 | 0.11 | 0.11 | 0.14 | 0.10 | 0.10 | 0.11 | 0.16 | 0.14 |
| Ethylbenzene |  | 0.07 | 0.24 | 0.09 | 0.07 | 0.37 | 0.22 | 0.18 | 0.27 | 0.48 | 0.11 | 0.10 | 0.08 |
| *Trans*-isoeugenol | Phenol (2) | 1.58 | 1.49 | 1.41 | 1.53 | 1.31 | 1.80 | 1.64 | 1.71 | 1.93 | 2.39 | 2.69 | 2.72 |
| 2-Propyl-phenol |  | 0.77 | 0.96 | 0.95 | 0.89 | 1.10 | 1.80 | 1.47 | 1.63 | 2.81 | 4.71 | 4.82 | 3.87 |
| Indole | heterocyclic compound (3) | 6396.67 | 6921.95 | 6845.62 | 7242.79 | 6427.10 | 7273.76 | 7013.04 | 7693.25 | 9724.78 | 10401.14 | 10314.91 | 10700.27 |
| 2-Pentylfuran |  | 0.35 | 0.47 | 0.44 | 0.49 | 0.70 | 0.54 | 0.67 | 0.46 | 0.84 | 0.97 | 1.29 | 0.96 |
| 2-Ethylfuran |  | 0.07 | 0.08 | 0.08 | 0.06 | 0.09 | 0.08 | 0.09 | 0.07 | 0.31 | 0.28 | 0.18 | 0.15 |
| (2-Nitroethyl)-benzene | Other (2) | 10.25 | 10.77 | 11.06 | 11.16 | 12.16 | 13.93 | 12.48 | 13.85 | 22.98 | 25.27 | 25.12 | 23.81 |
| Benzyl nitrile |  | 6.39 | 7.50 | 7.53 | 7.64 | 10.37 | 12.28 | 11.49 | 12.23 | 25.69 | 26.90 | 25.76 | 23.17 |

The 4 h residues (ck1) represented the residues of JGY after 4 h tea extraction of tea hydrolat; the 40 min residues (ck2) represented the residues of JGY after 40 min extraction of tea hydrolat; JGY represented the JGY tea; 1:3, 1:6, 1:9 represented the residues/tea after spraying the tea hydrolat when the ratio of the tea hydrolat to the tea leaves was 1:3, 1:6, 1:9, respectively.
